# Crystal structures of the NAD^+^-II riboswitch reveal two distinct ligand-binding pockets

**DOI:** 10.1093/nar/gkad102

**Published:** 2023-02-25

**Authors:** Xuemei Peng, Wenjian Liao, Xiaowei Lin, David M J Lilley, Lin Huang

**Affiliations:** Guangdong Provincial Key Laboratory of Malignant Tumor Epigenetics and Gene Regulation, Guangdong-Hong Kong Joint Laboratory for RNA Medicine, Sun Yat-Sen Memorial Hospital, Sun Yat-Sen University, Guangzhou, China; Medical Research Center, Sun Yat-Sen Memorial Hospital, Sun Yat-Sen University, Guangzhou, China; Guangdong Provincial Key Laboratory of Malignant Tumor Epigenetics and Gene Regulation, Guangdong-Hong Kong Joint Laboratory for RNA Medicine, Sun Yat-Sen Memorial Hospital, Sun Yat-Sen University, Guangzhou, China; Department of Urology, Sun Yat-Sen Memorial Hospital, Sun Yat-Sen University, Guangzhou, China; Guangdong Provincial Key Laboratory of Malignant Tumor Epigenetics and Gene Regulation, Guangdong-Hong Kong Joint Laboratory for RNA Medicine, Sun Yat-Sen Memorial Hospital, Sun Yat-Sen University, Guangzhou, China; Department of Urology, Sun Yat-Sen Memorial Hospital, Sun Yat-Sen University, Guangzhou, China; Cancer Research UK Nucleic Acid Structure Research Group, MSI/WTB Complex, The University of Dundee, Dow Street, Dundee DD1 5EH, UK; Guangdong Provincial Key Laboratory of Malignant Tumor Epigenetics and Gene Regulation, Guangdong-Hong Kong Joint Laboratory for RNA Medicine, Sun Yat-Sen Memorial Hospital, Sun Yat-Sen University, Guangzhou, China; Medical Research Center, Sun Yat-Sen Memorial Hospital, Sun Yat-Sen University, Guangzhou, China

## Abstract

We present crystal structures of a new NAD^+^-binding riboswitch termed NAD^+^-II, bound to nicotinamide mononucleotide (NMN), nicotinamide adenine dinucleotide (NAD^+^) and nicotinamide riboside (NR). The RNA structure comprises a number of structural features including three helices, one of which forms a triple helix by interacting with an A_5_ strand in its minor-groove, and another formed from a long-range pseudoknot. The core of the structure (centrally located and coaxial with the triplex and the pseudoknot) includes two consecutive quadruple base interactions. Unusually the riboswitch binds two molecules of ligand, bound at distinct, non-overlapping sites in the RNA. Binding occurs primarily through the nicotinamide moiety of each ligand, held by specific hydrogen bonding and stacking interactions with the pyridyl ring. The mode of binding is the same for NMN, NR and the nicotinamide moiety of NAD^+^. In addition, when NAD^+^ is bound into one site it adopts an elongated conformation such that its diphosphate linker occupies a groove on the surface of the RNA, following which the adenine portion inserts into a pocket and makes specific hydrogen bonding interactions. Thus the NAD^+^-II riboswitch is distinct from the NAD^+^-I riboswitch in that it binds two molecules of ligand at separate sites, and that binding occurs principally through the nicotinamide moiety.

## Graphical Abstract

**Graphical Abstract:**
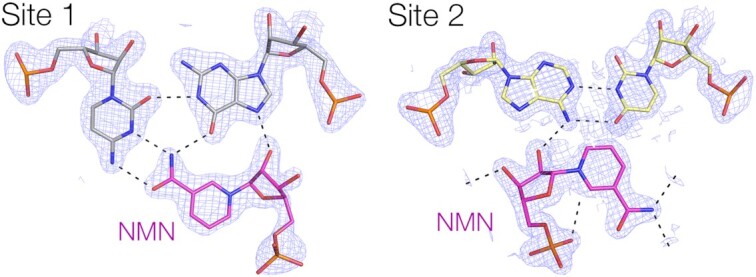
The two NMN binding sites observed in the NAD^+^-II riboswitch.

## INTRODUCTION

Riboswitches are regulatory elements generally found in the 5’ untranslated regions of bacterial genes whose conformation is altered by the binding of a small-molecule metabolite related to the function of the gene. The ligand-induced conformational switch leads to an altered level of transcription or translation of the downstream gene, either lowering (OFF switch) or raising (ON switch) the level of expression. One of the largest groups of riboswitches bind coenzymes, including the relatively recently-identified NAD^+^-binding riboswitches (1,2).

While most riboswitches comprise a single discrete RNA motif that binds one ligands, with the discovery of a large number of riboswitches binding a wide variety of ligands more complex examples have emerged. A number of tandem binding riboswitches have been found, binding glycine (3) and thiamine pyrophosphate (4), and one example is known of a triply-tandem riboswitch that binds cyclic-di-GMP (5). The tandem glycine-binding riboswitches exhibit cooperative binding, although this is clearly not essential as singlet examples are also known (6). Riboswitches binding distinct ligands can also occur in tandem, generating potential logic gates for gene regulation. These include *S*-adenosylmethionine plus coenzyme B_12_ (7) and phosphoribosyl pyrophosphate plus guanine (8) riboswitches. A different form of complexity exists where a single riboswitch binds two molecules of ligand. This includes the THF-I riboswitch that binds two molecules of folinic acid (9), the guanidine-II riboswitch comprising two interacting stem-loops where each binds a guanidine molecule (10), and the preQ_1_-I riboswitch that binds two mutually-stacked preQ_1_ ligands (11).

**Figure 1.**
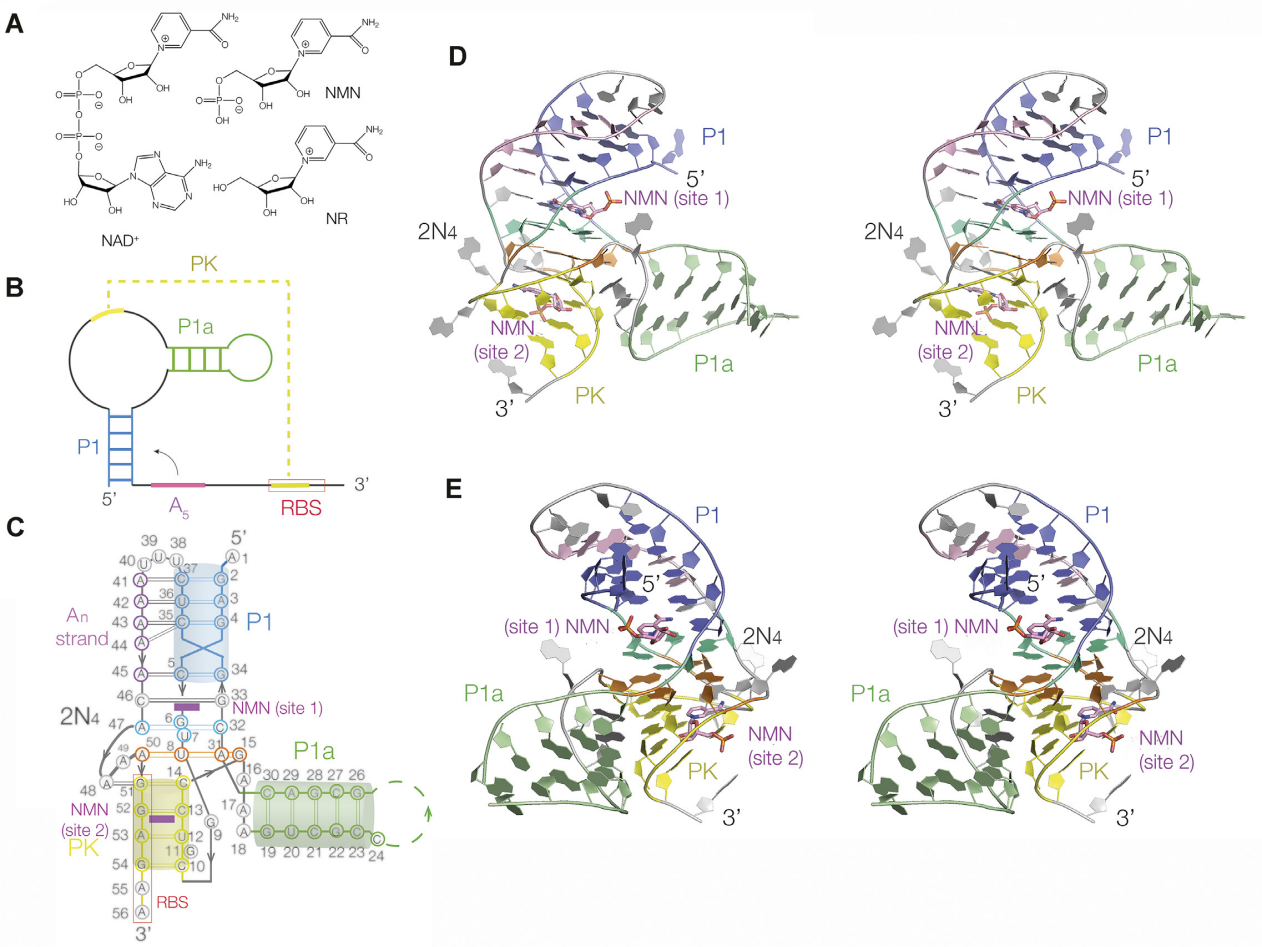
The overall structure of the NAD^+^-II riboswitch bound to nicotinamide mononucleotide. (**A**) The chemical structures of NAD^+^-II riboswitch ligands; NAD^+^, NMN and nicotinamide riboside (NR). (**B**) Scheme showing the secondary structure. The ribosome binding site (RBS) is boxed. (**C**) Scheme of the structure observed in the crystal structure of the riboswitch bound to NMN. The color scheme for the different structural elements is followed throughout the paper. The secondary structure elements are marked, and the ribosome binding site is boxed. (**D**, **E**) Parallel eye stereoscopic views from opposite sides of the NAD^+^-II riboswitch bound to NMN determined by X-ray crystallography.

The NAD^+^ riboswitch (1) (recently renamed the NAD^+^-I riboswitch) binds nicotinamide adenine dinucleotide (NAD^+;^ Figure 1A) to act as a translational OFF switch that comprises two rather similar domains. X-ray crystal structures (12,13) showed that the 5’ domain binds NAD^+^, but electron density for only the ADP end of the ligand could be observed, and structures of a variety of bound adenosine derivatives including ADP were also determined. Ren *et al.* (13) further showed that the 3’ domain bound NAD^+^ more weakly, and determined a crystal structure showing that the ligand bound in a similar manner by the ADP end of the coenzyme. Collectively these observations suggest interesting questions with regard to the possible modularity of this and similar riboswitches.

The NAD^+^-I structures provided no information on how the nicotinamide moiety of NAD^+^ might bind to RNA. However Breaker and colleagues (2) have discovered a second NAD^+^-binding riboswitch that responds primarily to the nicotinamide moiety. The new riboswitch is found in the 5’-untranslated region of the *pnu*C gene that encodes a putative transporter of nicotinamide riboside in a range of bacteria including *Streptococcus* and *Haemophilus influenzae*. Its proposed secondary structure includes a pseudoknot that overlaps the ribosome binding site (Figure 1B), suggesting that it acts as a translational OFF switch. The motif has been named the NAD^+^-II riboswitch. In-line probing indicated that the *pnu*C RNA alters its conformation on binding either NAD^+^ or nicotinamide mononucleotide (NMN), suggesting that this RNA primarily binds to the nicotinamide moiety. Furthermore Panchapakesan *et al.* found that NMN was bound to the RNA three-fold more tightly than was NAD^+^. This is in marked contrast to NAD^+^-I that binds the adenosine moiety of NAD^+^ (12,13), so that in terms of binding specificity the NAD^+^-I and NAD^+^-II riboswitches are complementary.

We have determined crystal structures of the NAD^+^-II riboswitch bound to the ligands NMN (Figure 1A) and NAD^+^ at good resolution. We find that the new riboswitch binds two molecules of NMN or NAD^+^ ligand, but that in contrast to the NAD^+^-I riboswitch binding to each ligand occurs primarily through the nicotinamide moiety.

## MATERIALS AND METHODS

### Riboswitch ligands

NAD^+^ (N1636), NMN (N3501) and NR (SMB00907) for crystallization were all obtained from Sigma. For gel electrophoretic analysis NMN (B886299) and NADH (N814671) were obtained from Macklin Biochemical Technology Co., Ltd (Shanghai, China), and ADP (A610016) from Sangon Biotech Co., Ltd (Shanghai, China).

### Synthesis of RNA

Full-length and split NAD^+^-II riboswitch sequences were designed based on the *pnu*C motif from *Streptococcus parasanguinis*. The full-length sequences were obtained by *in vitro* transcription, and the split sequences were made by chemical synthesis. All sequences are written 5′ to 3′.

RNA 5–24:AGAGCGUUGCGUCCGAAAGU(BrC)GCC

RNA 3–30:GCGACACGGCUCUUUAAAAACAAAAGGAGA

RNA 3–31:GCGACACGGCUCUUUAAAAACAAAAGGAGAA

RNA 55:GCGGCGUUGCGUCCGAAAGUCUAAACAGACACGGCCGCUUAAAAACAAAAGGAGA

### Chemical synthesis of RNA

RNA oligonucleotides were synthesized using ***t***-BDMS phosphoramidite chemistry (14) as described in Wilson *et al.* (15), implemented on an Applied Biosystems 394DNA/RNA synthesizer. RNA was synthesized using ribonucleotide phosphoramidites with 2′O-*tert*-butyldimethyl-silyl (*t*-BDMS) protection (16,17) (Link Technologies). Oligonucleotides containing 5-bromocytidine (ChemGenes) were deprotected in a 25% ethanol/ammonia solution for 36 h at 20°C. All oligoribonucleotides were redissolved in 100 μl of anhydrous DMSO and 125 μl triethylamine trihydrofluoride (Sigma-Aldrich) to remove *t*-BDMS groups, and agitated at 65°C in the dark for 2.5 h. After cooling on ice for 10 min, the RNA was precipitated with 1 mL of butanol, washed once with 70% ethanol and suspended in double-distilled water. RNA without bromine modification was purchased from Accurate Biotechnology (Hunan, China) Co., Ltd.

### Synthesis of RNA by transcription

To facilitate *in vitro* transcription, the starting nucleotides of the original sequences were changed to GCG, HDV ribozyme was used to generate homogenous 3' ends. DNA templates containing a T7 RNA polymerase promoter for *in vitro* transcription were synthesized using the polymerase chain reaction (PCR). *in vitro* transcription was carried out at 37°C for 5–6 h, followed by purification by gel electrophoresis in 10% denaturing (7 M urea) polyacrylamide. The gel was visualized with ultraviolet light, the targeted RNA was then excised and electroeluted in 45 mM Tris-borate, 1 mM EDTA buffer for 12 h at 150 V. The eluted RNA was precipitated with isopropanol and washed once with 75% ethanol, then RNA was dissolved with double-distilled water to 10 mg/ml for crystallization.

### Crystallization, structure determination and refinement

A solution containing 0.6 mM RNA 5–24 and 0.6 mM RNA 3–30 or 3–31 or 0.6 mM full-length NAD^+^-II riboswitch RNA (55 nt) in 5 mM HEPES (pH 7.5), 100 mM KCl, 5 mM MgCl_2_ (HKM buffer) was heated to 65°C for 5 min, the ligand (NAD^+^, NMN or NR) was added to a final concentration of 5 mM after the RNA solution cooled at room temperature for 5 min. RNA sequence and crystallization conditions are summarised in Supplementary Table S1. Crystallization was performed by mixing 0.2 μl of the RNA-ligand complex with 0.2 μl of reservoir solution using sitting drop vapour diffusion at 18°C. Well-diffracting crystals of RNA species 5–24 and 3–30 (Supplementary Table S1) with NMN and 5–24 and 3–31 with NR grew from the 2 M ammonium sulfate after 7 days. The crystals were transferred into mother liquor with an additional 30% glycerol, and then flash frozen by mounting in nylon loops and plunging into liquid nitrogen. Well-diffracting crystals of full-length NAD^+^-II riboswitch RNA with NAD^+^ or NMN grew from 0.012 M NaCl, 0.08 M KCl, 0.04 M Na cacodylate trihydrate (pH 5.5), 45% v/v (+/−)-2-methyl-2,4-pentanediol, 0.02 M hexammine cobalt (III) chloride after 7 days. Crystals were flash-frozen in liquid nitrogen before data collection. X-ray diffraction data were collected on beamline BL02U1 or BL19U1 at Shanghai Synchrotron Radiation Facility (SSRF) and processed with XIA2 or XDS (18). The resolution cutoff for the data was determined by examining by CC1/2 and the density map (19). The 2.23 Å structure deposited as 8HB1 was determined by Br-SAD by AutoSol in PHENIX suite. The mean overall figure of merit for all reflections between 47.3 and 2.3Å is 0.192. 35 of 55 RNA residues could be built automatically by AutoBuild in the PHENIX suite. The model was adjusted manually using Coot and subjected to several rounds of adjustment and optimization using Coot, phenix.refine, and PDB_REDO (20). All other structures were determined by molecular replacement using PHASER (21) with PDB 8HB1 or 8HBA. The translation function Z scores (TFZ) and log-likelihood gains (LLG) are reported in Supplementary Table S1. Model geometry and the fit to electron-density maps were monitored with MOLPROBITY (22) and the validation tools in Coot. Simulated annealing omit maps were calculated by Composite omit map in the PHENIX suite using the method anneal. Atomic coordinates and structure factor amplitudes have been deposited with the PDB with accession code as listed in Supplementary Table S2, together with the statistics of crystal diffraction data and structure refinement.

### Gel electrophoretic analysis of ligand binding

Ligand binding ability was analysed by polyacrylamide gel electrophoresis in a two-strand system. Hybridization of two oligonucleotides was carried out by denaturing at 65°C for 5 min in 25 mM Tris, 192 mM glycine (pH 8.3), 1 mM MgCl_2_ with or without 1 mM NMN or other ligands followed by slow cooling. Electrophoresis was performed in 20% polyacrylamide gels at temperatures between room temperature at 42°C with buffer recirculation at 100 V for 2 h with or without 1 mM NMN added to the gel and running buffer. After electrophoresis RNA was visualized by UV shadowing. Sequences (all written 5’ to 3’) for native gel electrophoretic analysis were:

RNA 5–15:AGAGCGUUGCGUCCG

RNA 5–15-C5U:AGAGUGUUGCGUCCG

RNA 5–15-C10U:AGAGCGUUGUGUCCG

RNA 3–25:ACGGCUCUUUAAAAACAAAAGGAGA

RNA 3–25-G33A:ACAGCUCUUUAAAAACAAAAGGAGA

RNA 3–25-C46U:ACGGCUCUUUAAAAAUAAAAGGAGA

RNA 3–25-A49G:ACGGCUCUUUAAAAACAAGAGGAGA

RNA 3–25-A53G:ACGGCUCUUUAAAAACAAAAGGGGA

## RESULTS

### Crystallization of the NAD^+^-II riboswitch

We have used two forms of the NAD^+^-II riboswitch in these studies (Supplementary Figure S1). The first comprised two strands made by chemical synthesis, so that the terminal loop of helix P1a was not present. 5-Bromocytidine was incorporated at C21 in order to determine the phases by SAD. The second was made as a single strand by transcription. Three ligands have been studied; NAD^+^, nicotinamide adenine dinucleotide (NMN) and nicotinamide riboside (NR) (Figure 1A). Breaker *et al.* (2) have shown that NMN binds about 3-fold more tightly to the RNA than NAD^+^. Sequences and conditions used in crystallization are summarised in Supplementary Table S1.

### The structure of the NAD^+^-II riboswitch bound to nicotinamide mononucleotide

We obtained three datasets for the NAD^+^-II riboswitch bound to NMN. The two-strand form of the riboswitch bound to NMN crystallised in space group *P*3_2_21 and diffracted to a resolution of 2.23 Å; these data were used to determine the structure by Br-SAD. A single stranded form of the NAD^+^-II riboswitch bound to NMN crystallised in space group *I*4_1_22 and diffracted to a resolution of 2.30 Å. Lastly, an additional two-strand form of the NAD^+^-II riboswitch bound to NMN crystallised in space group *P*3_2_21 and diffracted to a resolution of 1.67 Å. The RMSD values between these three structures were all below 0.6 Å, so we use the highest resolution structure for subsequent discussion.

The structure is shown schematically in Figure 1B, C, and opposite views of the 3D structure shown in Figure 1D, E. All the inter-nucleotide molecular interactions are shown in graphical form in Supplementary Figure S2. Four 5*’* nucleotides (G2 through to C5) form the P1 stem, a duplex that forms a minor groove triplex with the five-adenine (A_5_) strand. This then passes into the 2N_4_ region (so called because it contains two tetra-nucleobase interactions; G6 to U8) comprising a number of multiple-base interactions discussed below. The strand then loops around via an extended guanine (G9) to the distal end of the long-distance pairing interaction forming the pseudoknot PK helix (C10–C14). On exiting from the PK helix the strand contributes the guanine nucleobase (G15) of the lower quadruple-base interaction, followed by a three-adenine connecting region (A16–A18) before entering the P1a helix (G19–C23). In the complete riboswitch P1a is a stem loop, but in our construct the loop is not present. The strand then passes back down the P1a helix (G26–C30), then forming a second strand of the 2N_4_ region (A31–G33) before forming the second strand of the P1 helix (G34–C37). At the end of P1 the strand turns around via a three-uridine run and then the A_5_ section forms the minor groove triple helix with P1 (A41–A45). It then forms the third strand of the 2N_4_ region (C46–A50) and finally forms the second strand of the PK helix G51–G54). The P1 triplex, the 2N_4_ region and the PK helix are coaxial in the folded RNA structure, with uninterrupted base stacking throughout. The axis of the P1a helix lies at right angles to that of the P1-2N_4_-PK helices, at the level of the 2N_4_ section.

### The P1, P1a and PK helices

**Figure 2.**
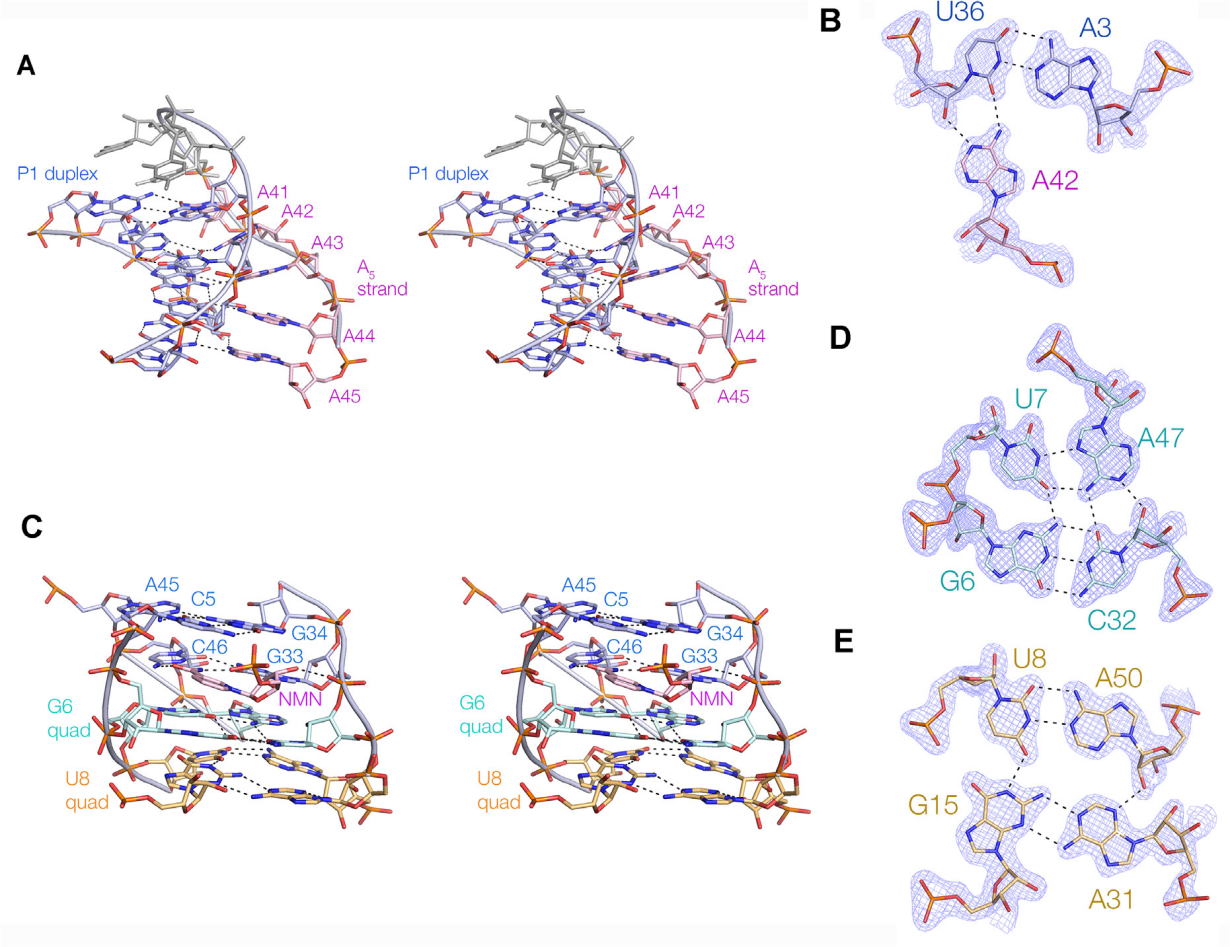
Structural elements in the NAD^+^-II riboswitch bound to nicotinamide mononucleotide. (**A**) Parallel eye stereoscopic view of the P1 triple helix. This is formed by the A_5_ strand (pink) interacting with the minor groove of the P1 duplex (blue). (**B**) An example of one of the triple interactions, showing the hydrogen bonding between the Watson–Crick edge of the A42 nucleobase with the sugar edge of U36 of the A3:U36 base pair. The electron density shows the simulated annealing omit map contoured at 2σ. (**C**) Parallel eye stereoscopic view of the 2N_4_ core of the riboswitch that contains two quadruple base interactions. The G6-containing quadruple base interaction (G6 quad) is colored cyan and the U8-containing quadruple base interaction (U8 quad) is colored orange. The NMN ligand bound at site1 in this region is colored magenta. (**D** and **E**) The two quadruple base interactions, G6:U7:A47:C32 (D) and U8:A50:A31:G15 (E). The electron density shows the simulated annealing omit map contoured at 2σ.

P1, P1a and PK are all paired by standard *cis*-Watson-Crick base pairing. In addition the P1 helix binds the A_5_ strand in its minor groove (Figure 2A, B). The first two triples (that include G2 and A3 respectively) are formed with G:C and A:U pairs, but they are isosteric, with hydrogen bonds formed between the N1 and N6 of the adenines of the third strand (A41 and A42 of A_5_) with O2’ and O2 of the pyrimidine (C37 and U36). The third triple interaction is more complicated, involving two of the adenine nucleotides. The A43 makes two hydrogen bonds to G4 (A43 N6 to G4 N3 and G4 N2 to A43 N1), but A44 also interacts, making equivalent hydrogen bonds with C35 as in the first two triple base interactions. Finally in the forth triple the base pair is reversed relative to the first three, and in this case the adenine (A45) forms hydrogen bonds to both pairing partners, i.e. its N1 and N6 are bonded to G34 N2 and C5 O2’ respectively.

### The structure of the 2N_4_ region

The 2N_4_ region may be regarded as the core of the riboswitch; its structure is shown in Figure 2C. We define the 2N_4_ region to begin at the G33:C46 base pair that binds one of the NMN ligands that we discuss below. This is immediately followed by two planes of four coplanar nucleobase interactions. The first quadruple base interaction (Figure 2D) comprises G6-(*cis* Watson–Crick)-C32-(*cis* sugar-Watson–Crick)-A47-(*cis* Hoogsteen–Watson–Crick)-U7. The final hydrogen bond that completes the circle is donated from G6 N2 to U7 O4. The second quadruple base interaction (Figure 2E) comprises A50-(*trans*-Watson–Crick)-U8-(*trans* Hoogsteen–Watson–Crick)-G15-(*trans* sugar-Watson–Crick)-A31. There is no base-base interaction between A31 and A50, but the circle is completed by a hydrogen bond donated from A50 O2’ to A31 N3.

### The binding of NMN ligands to the RNA

**Figure 3.**
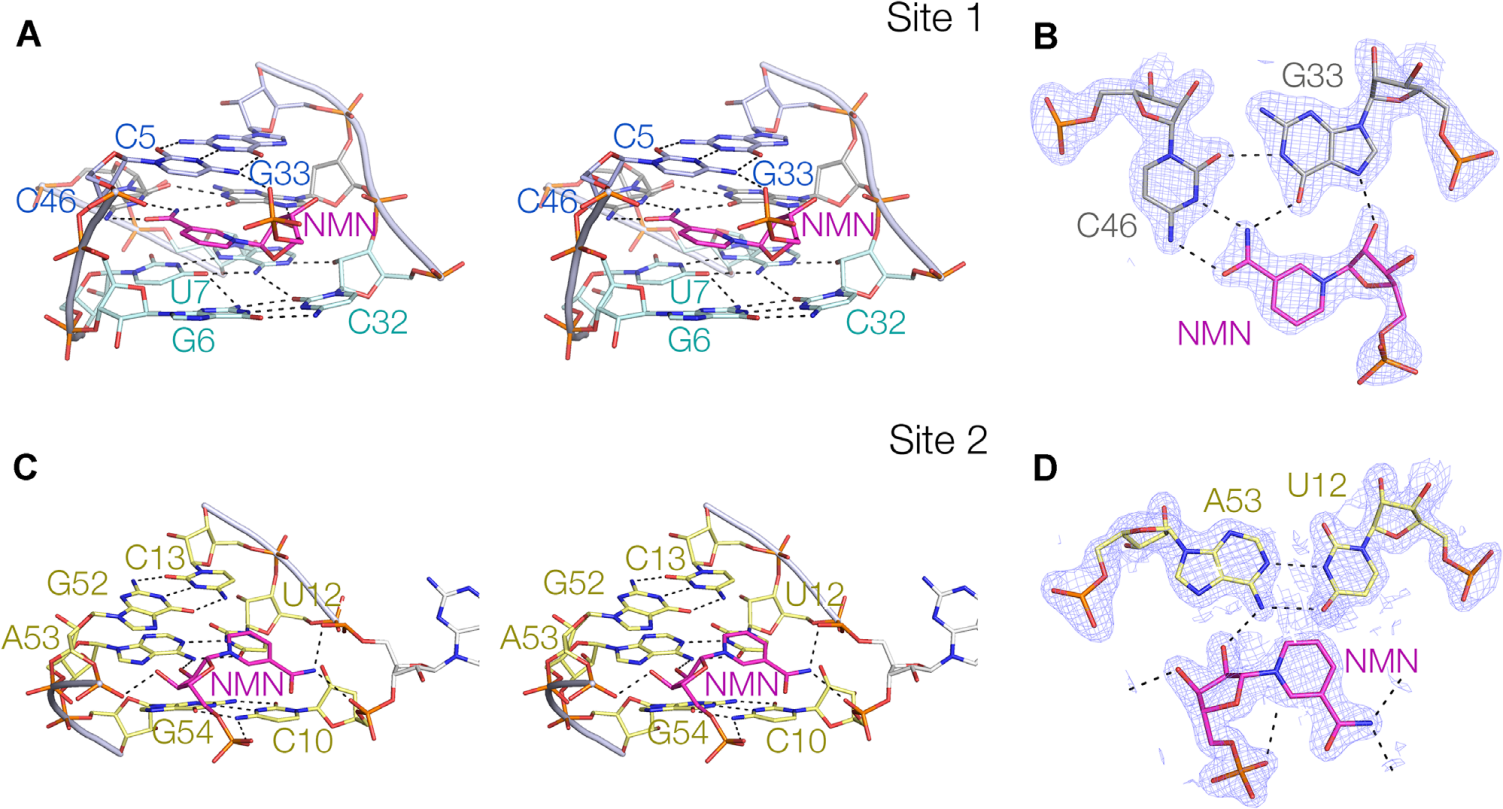
The two nicotinamide binding sites of the NAD^+^-II riboswitch bound to nicotinamide mononucleotide. (**A**) Parallel eye stereoscopic view of the binding site 1 within the 2N_4_ region. The pyridyl ring of the NMN is coplanar with the G33:C46 base pair, and stacked between the C5:G34:A45 triple interaction and the G6-containing quadruple base interaction. The phosphate of the NMN ligand accepts a hydrogen bond from C5 N4 in the triple interaction. (**B**) The in-plane interaction between the NMN and the G33:C46 base pair. The electron density simulated annealing omit map is shown contoured at 2σ. (**C**) Parallel eye stereoscopic view of the binding site 2 in the major groove of the PK helix. (**D**) The interaction between the nicotinamide and the G12:A53 base pair. The electron density simulated annealing omit map is shown contoured at 2σ. For clarity the electron density has not been shown for C10.

We observe two molecules of nicotinamide bound at distinct sites within the riboswitch RNA (Figure 3). Both NMN ligands were well defined by the electron density map. We term the binding sites site 1 that is within the 2N_4_ region (Figure 3A, B), and site 2 within the PK helix (Figure 3C, D).

At the site 1 site the NMN effectively forms an integral part of the 2N_4_ structure (Figure 3A). The NMN is adjacent to, and coplanar with, the G33:C46 base pair on its major groove edge (Figure 3B), and stacked on both faces. The pyridyl ring is stacked on the G6 nucleobase of the G6:C32:A47:U7 quadruple base on one side, and C5 of the C5:G34:A45 triple on the other. The positive charge on the ring nitrogen atom is thus involved in a cation–π interaction with the nucleobase of G6 in particular. This enables the riboswitch to distinguish between NAD^+^ (positively charged) and NADH (neutral) in a form of electrostatic discrimination akin to the selective binding of SAM versus SAH in the SAM V riboswitch (23). The nicotinamide moiety is extensively hydrogen bonded to the G33:C46 base pair, with the amide O accepting a bond from C46 N4, and the amide N donating hydrogen bonds to C46 N3 and G33 O6. The NMN ribose O2’ hydroxyl group donates a hydrogen bond to G33 N7. Furthermore, one of the ligand phosphate oxygen atoms accepts a hydrogen bond from C5 N4. Altogether five hydrogen bonds are made between the NMN ligand and the RNA. Binding the ligand holds the G33:C46 base pair in an unusual structure, forming a *cis* Watson–Crick base pair connected by a single hydrogen bond donated by G33 N1 to C46 O2. We would expect this conformation to be unstable compared to a standard Watson–Crick base pair, and the binding of the NMN clearly stabilises the alternative structure.

A second NMN molecule is bound at site 2 in the major groove of the PK helix (Figure 3C). The pyridyl ring is coplanar with the C13:G52 base pair (Figure 3D), although there are no direct hydrogen bonds formed with either nucleobase. The NMN ribose O2’ is hydrogen bonded to N6 of A53, and one oxygen atom of the ligand phosphate accepts a hydrogen bond from C10 N4. The remaining hydrogen bonds are made with backbone non-bridging oxygen atoms. The amide N donates one proton to the *pro*R non-bridging oxygen of G9 that links the 2N_4_ and PK helices, and the other to the *pro*R non-bridging oxygen of C10. The NMN ribose O3’ donates a proton to the *pro*R non-bridging oxygen of G52. Altogether five hydrogen bonds are made between the NMN ligand and the RNA. The pyridyl ring is poorly stacked, and in contrast to the NMN at site 1 the pyridyl ring N does not appear to be in a position to contribute to significant cation-π stacking.

### The binding of NAD^+^ to the NAD^+^-II riboswitch

**Figure 4.**
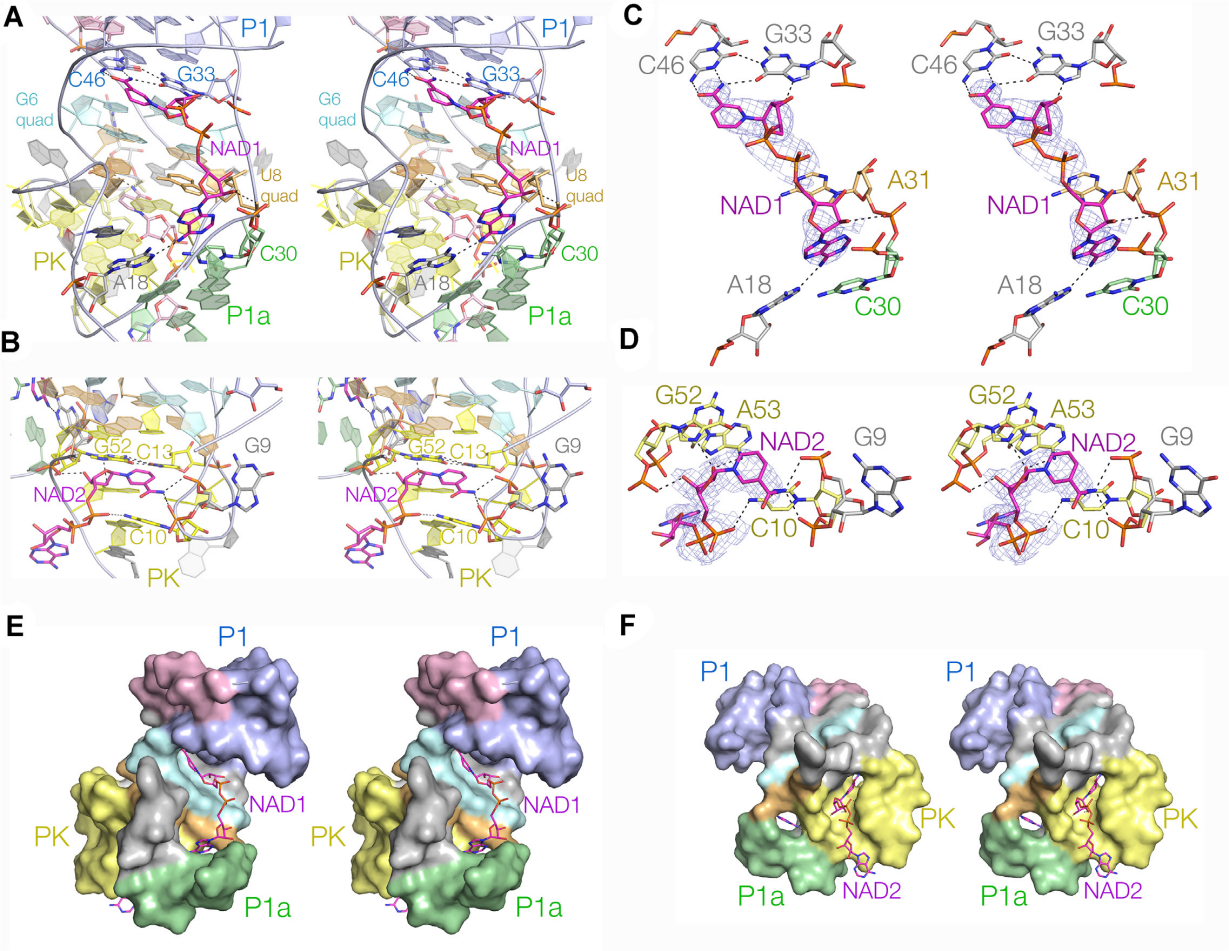
The structure of the NAD^+^-II riboswitch bound to NAD^+^. Parallel eye stereoscopic views of the RNA and ligand binding sites. (**A**) View of the P1a side of the riboswitch showing the binding of NAD^+^ at site 1. At the top the nicotinamide binds in plane to the G33:C46 base pair analogously to NMN, the diphosphate chain passes over the two quadruple base interactions, and the adenine end interacts with C30 and A18 at the end of the P1a helix. (**B**) View into the major groove of the PK helix showing the binding of NAD^+^ at site 2. The NAD^+^ nicotinamide is bound in plane with the C13:G52 base pair analogously to that of NMN. (C and D) View of the trajectory of the site 1- (**C**) and site 2- (**D**) bound NAD^+^ molecules, showing the hydrogen bonds formed with the RNA. The electron density simulated annealing omit maps for the NAD^+^ are shown contoured at 1σ. (**E**) View of NAD^+^ bound to site 1 with the RNA shown in space-filling mode. The nicotinamide and adenine moieties are bound in cavities in the RNA, while the connecting diphosphates traverse the two quadruple base interactions (cyan and orange). (**F**) View of NAD^+^ bound to site 2 with the RNA shown in space-filling mode. The nicotinamide moiety is bound within the major groove, while the adenine end emerges from the groove into the solvent.

A one-strand form of the NAD^+^-II riboswitch was co-crystallised with NAD^+^ yielding crystals in the space group *P*2_1_2_1_2_1_, and the structure determined at a resolution of 2.64 Å by molecular replacement using the two-strand NMN-bound structure discussed above as the search model. The RNA structure of the riboswitch bound to NAD^+^ superimposes very well with that bound to NMN (Supplementary Figure S3), with an RMSD of 0.582 Å. The structure contains two bound NAD^+^ ligands (Figure 4A, B), with their nicotinamide moieties bound essentially identically to the NMN in the two-strand structure discussed above.

The electron density map (Figure 4C) for the nicotinamide moiety and diphosphate bound at site 1 is very good, but poorer for the adenosine moiety. This suggests some mobility for the adenosine end of the NAD^+^. But nevertheless, the adenine predominantly locates in a pocket at the end of the P1a helix. The adenine nucleobase is stacked on that of C30 at the end of P1a, and within the pocket its N6 donates a hydrogen bond to N1 of A18. In effect the NAD^+^ adenine and A18 form a *trans* Hoogsteen-Watson-Crick base pair stacked on the end of the P1a helix. Although its ribose is not well defined by the electron density, its O2’ probably donates a hydrogen bond to the *pro*R non-bridging phosphate oxygen of A31 in the U8 quadruple base interaction. The NAD^+^ ligand binds to the riboswitch in an extended conformation (the nicotinamide and adenine moieties are separated by 14 Å), on the major groove side of the 2N_4_ region, with nicotinamide and adenine ends located in well-separated cavities in the RNA (Figure 4E).

The positions of the nicotinamide moiety and diphosphate for the ligand bound at site 2 are well defined, but the position of the adenine nucleoside (particularly the ribose) is poorly defined (Figure 4D). The nicotinamide moiety binds in the same manner as that in the NMN-bound structure, in plane with the C13:G52 base pair in the major groove of the PK helix. The *pro*S non-bridging oxygen atom of the phosphate proximal to the nicotinamide accepts a hydrogen bond from the N4 of C10. The trajectory of the diphosphates indicates that the adenine moiety emerges from the PK major groove, but the adenine appears to make no interactions with the RNA (Figure 4B, F).

### The binding of nicotinamide riboside to the NAD^+^-II riboswitch

We have determined the structure of the two-strand version of the NAD^+^-II riboswitch bound to NR at a resolution of 2.87 Å (Supplementary Figure S4). The overall structure of the riboswitch (Supplementary Figure S4A) is essentially identical to the structures bound to NMN and NAD^+^ (Supplementary Figure S3), including the three helical sections and the 2N_4_ core. The RNA structure of the riboswitch bound to NR superimposes very well with that bound to NMN, with an RMSD of 0.365 Å. As with the other complexes, two molecules of NR are observed bound to the RNA, at the same two sites observed for NMN and NAD^+^. At site 1 the nicotinamide moiety of NR is bound in plane with the G33:C46 base pair (Supplementary Figure S4B), and hydrogen bonded in the same manner. However, because NR lacks the phosphate group there can be no interaction with C5. At site 2 the nicotinamide moiety of NR is bound in plane with the C13:G52 base pair in the major groove of the PK helix (Supplementary Figure S4C), and hydrogen bonded equivalently to that of NMN and NAD^+^. As with the site 1 because NR lacks the phosphate group there can be no interaction with C10 at the lower end of the PK helix.

### Ligand binding investigated by gel electrophoresis

**Figure 5.**
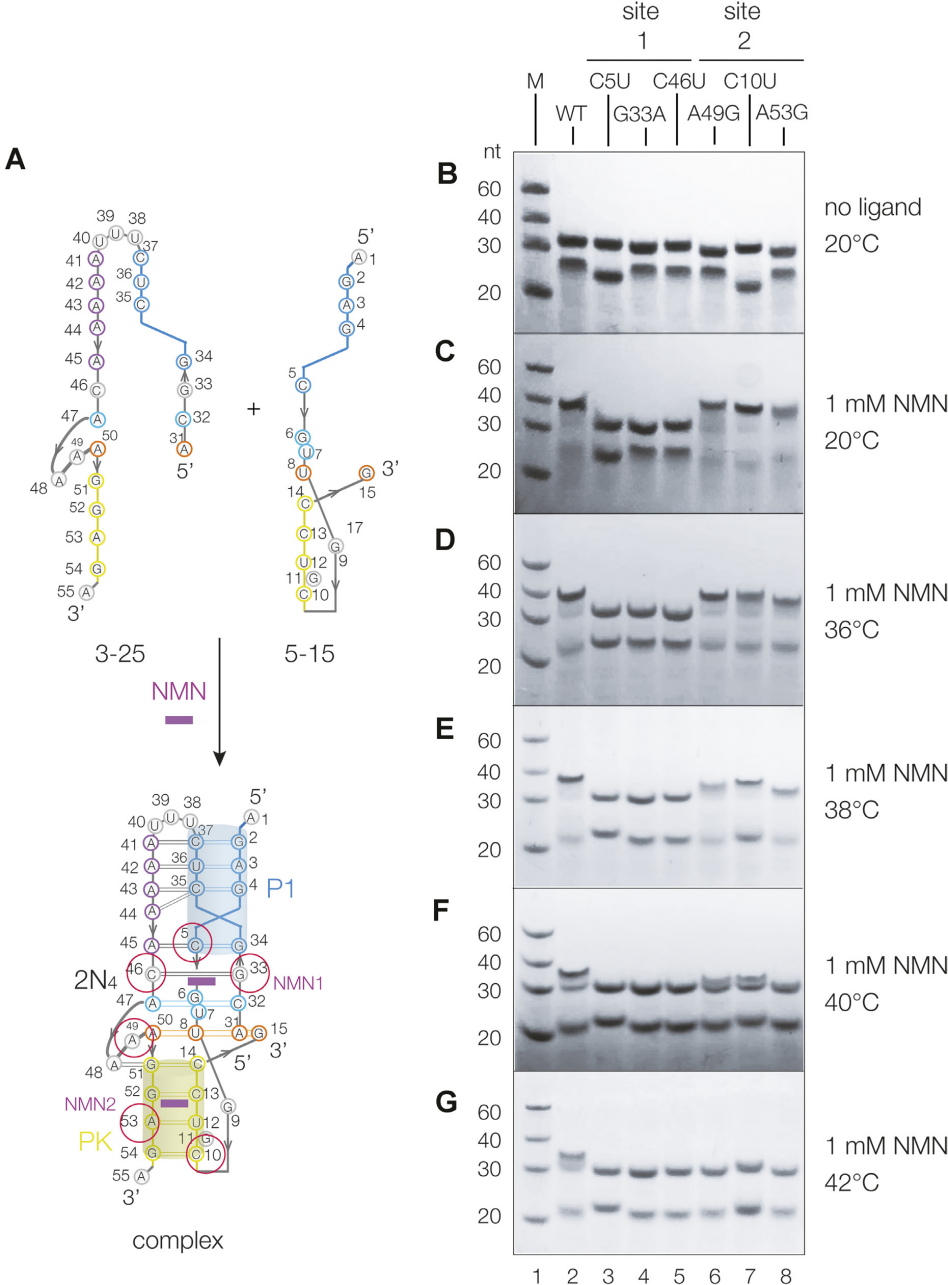
Ligand binding analysed by polyacrylamide gel electrophoresis. (**A**) Scheme showing the principle of the riboswitch assembly. The NAD^+^-II riboswitch has been divided into two individual strands, the 5’ section 1–15 nt (5–15) and the 3’ section 31–55 nt (3–25). In the presence of NMN ligand these assemble into a form of the NAD^+^-II riboswitch lacking helix P1a. Mutations were made in individual strands. G33A, C46U, A49G and A53G are in strand 3–25, while C5U and C10U are in strand 5–15. C5U, G33A and C46U are located within site 1, while A49G, C10U and A53G are in site 2. (**B–G**) Polyacrylamide gel electrophoresis of wild-type and mutant NAD-II riboswitches in the absence of ligand (B) or the presence of 1 mM NMN (C-G). Electrophoresis was performed at room temperature (B and C), 36°C (D), 38°C (E), 40°C (F) or 42°C (G). Tracks 1, a mixture of marker DNA fragments of 20, 30, 40 and 60 nt to provide a frame of reference; 2, wild-type sequence; 3, G5U; 4, G33A; 5, C46U; 6, A49G; 7, C10U; 8, A53G.

We have explored the requirement for RNA-ligand contacts identified in our crystal structures on RNA folding by means of polyacrylamide gel electrophoresis. We divided the RNA into two component oligonucleotides termed 5–15 (the 5’ strand of 15 nt) and 3–25 (the 3’ strand of 25 nt) that should assemble into the NAD^+^-II riboswitch lacking helix P1a (Figure 5A). Hybridization of this form into a stable complex is completely dependent on bound ligand and is sensitive to mutations that interfere with ligand binding.

Upon incubation of a mixture of the two unmodified strands they migrate as discrete oligonucleotides in the absence of ligand at room temperature (Figure 5B; track 2), with no slower species indicative of complex formation. However, when these oligonucleotides were incubated and electrophoresed in the presence of 1 mM NMN only a single band with retarded mobility was observed (Figure 5C; track 2). Individually the component strands exhibited no significant change in mobility on addition of 1 mM NMN (Supplementary Figure S5A).

We explored the effect of mutation at the two ligand binding sites, designed to interfere with ligand contacts observed in the crystal structures. For site 1 we have studied three mutations, C5U (track 3), G33A (track 4) and C46U (track 5) each of which will break ligand-nucleobase hydrogen bonds to NMN (see Figure 3). Each mutation resulted in the complete prevention of complex formation at room temperature (Figure 5C). For site 2 we also analyzed three mutation, A49G (track 6), C10U (track 7) and A53G (track 8). C10 and A53 make direct ligand-nucleobase hydrogen bonds in the crystal, while A49 is close to the binding site. Unlike the site 1 mutations, changes at site 2 do not prevent complex formation at room temperature (Figure 5C), although the band corresponding to the complex with A53G is more diffuse than those for the other variants. From these results we conclude that NMN binding at site 1 is more critical than that at site 2, and more sensitive to disruption of ligand-RNA hydrogen bonding.

To investigate further the sensitivity of ligand-induced folding to mutation in the two sites we repeated the electrophoretic analysis at a series of increasing temperatures to 42°C (Figure 5D–G). The higher temperatures reveal the sensitivity to site 2 mutations. At 38°C and above, where the unmodified riboswitch is still substantially folded, there is a significant fraction of unfolded RNA (i.e. two faster-migrating species visible) for the site 2 mutants, and by 42°C these are completely unfolded whereas the unmodified riboswitch is still significantly folded. The most sensitive of the site 2 mutants is A53G; in the crystal A53 N6 donates a hydrogen bond to NMN O2’. We conclude that NMN binding at site 1 is more important than at site 2, but nevertheless site 2 binding does contribute to the stability of the ligand-bound form.

We repeated the electrophoretic analysis for two other potential ligands, i.e. ADP and NADH (Supplementary Figure S5B). For both compounds no complex formation was observed at room temperature for the unmodified nor any mutant riboswitch.

## DISCUSSION

We have determined a number of crystal structures of the NAD^+^-II riboswitch using either two- or one-strand RNA constructs, in trigonal, tetragonal or orthorhombic crystallographic space groups, and with three different ligands bound. The structures are in excellent correspondence with one-another, superimposing with pairwise RMSD values of 0.6 Å or lower. All the structures exhibit two non-overlapping ligand binding sites located on opposite sides of the RNA, where the nicotinamide moieties are bound with high selectivity.

The three-dimensional RNA structures observed by crystallography are in full agreement with the secondary structure proposed by Breaker and colleagues (2), with the three helical domains P1, P1a and the long-range pseudoknot PK. In addition we find that the P1 helix forms a triple helix by incorporating the A_5_ sequence into its minor groove, forming a series of triple-base interactions. The P1 triple helix is continuous with the complex core region we term 2N_4_, that includes the two quadruple base interactions. The lower quadruple base interaction is stacked on the PK helix, and the P1 triplex, the 2N_4_ core and the PK helix are coaxial. The observed RNA structure is consistent with the in-line probing data of Panchapakesan et al (2). P1, P1a and PK all correspond to regions of low in-line cleavage. In addition two regions of enhanced cleavage were observed; A16 and A49 are both extra-helical, and thus these regions are likely to be more mobile and thus able to sample an in-line conformation. Indeed, these two regions exhibit the greatest structural variation between the different ligand-bound complexes (Supplementary Figure S3).

We consistently observe two binding sites within the NAD^+^-II riboswitch for all the ligands studied. NMN, NR and the nicotinamide moiety of NAD^+^ were observed making multiple hydrogen bonding interactions at sites 1 and 2. At site 1 the pyridyl ring is coplanar with the G33:C46 base pair, and stacked between the C5:G34-A45 triple, and the G6:C32:A47:U7 quadruple interaction, making a probable cation-π interaction with G6. This is likely important in allowing the riboswitch to distinguish between NAD^+^ and the neutral NADH. We note that the M1 and M2 mutations of Panchapakesan et al (2) that were found to prevent ligand binding are both nucleotides that are located in the quadruple base interactions observed in our structure. On binding at site 1 the nicotinamide moiety essentially becomes an integral part of the helical structure of the RNA, stabilizing the overall RNA structure. At the site 2 the pyridyl ring is coplanar with the C13:G52 base pair in the major groove of the PK helix.

Thus the binding of both ligands likely contributes to the stability of the overall fold of the ligand-bound RNA, and we found that mutations at both sites destabilized the complex. The riboswitch likely functions by occluding the ribosome binding site due to the formation of the PK helix, but that should form cooperatively with the 2N_4_ core of the structure. The overall stability should therefore be dependent on ligand binding at both sites, although the binding isotherms that Panchapakesan et al (2) generated from their in-line probing data provide no evidence of cooperativity in the presence of high concentration of Mg^2+^ ions.

Ligand binding by the NAD^+^-II riboswitch is almost the diametrical opposite of that of the NAD^+^-I riboswitch, where only binding of the adenosine moiety of NAD^+^ was observed in the crystal (12,13). Moreover, the NAD^+^-I riboswitch bound the diphosphate group of NAD^+^ and ADP by direct bonding to bound divalent metal ions within the RNA structure (12). By contrast the NAD^+^-II riboswitch predominantly binds the nicotinamide moiety, although the adenine nucleobase of the site 1 ligand is bound (probably more weakly) in a pocket at the end of the P1a helix. Thus the two riboswitches make an interesting comparison, in binding the NAD^+^ coenzyme in completely different ways.

While this paper was being reviewed another structure of the NAD-II riboswitch was published by Ren and co-workers (24). In most respects the structure was closely similar to those we have determined, but ligand binding was not observed at the equivalent of our site 2. However, in that structure two riboswitch molecules interacted to form a dimer, and the contact involved a loop-loop interaction in place of the pseudoknot helix. It is an open question whether or not that would prevent NMN and NAD^+^ binding to that region, but some of the important ligand-RNA contacts we observe would not be possible.

This raises the question of whether or not site 2 in our structure is functionally significant. We have observed binding at site 2 in crystals of three different space groups, and with NMN, NAD^+^ and NR bound. In each case binding to site 2 occurs with the nicotinamide portion of the coenzyme, suggesting that it has evolved to bind specifically. Bioinformatic analysis (2) (Supplementary Figure S6) shows that both site 1 and site 2 nucleotides are strongly conserved. In site 2 G9, C10, U12, C13, C14, A48, G51, G52, A53 and G54 are all conserved; these include all the nucleotides making specific interactions with NMN. Interestingly nucleotides 11 and 16 are not conserved; both are extended into the solvent in our structure, making no contacts with the riboswitch or ligand.

Binding site 2 is located in the major groove of the PK helix. Formation of the pseudoknot will prevent the ribosome binding site of the mRNA from interacting with the rRNA to initiate translation, so ligand binding at site 2 occurs at functionally the most important region of the riboswitch. Binding is much weaker than that at site 1, so that cooperative binding would not necessarily be observed. It is probable that site 2 binding will occur in riboswitch that is already pre-folded by binding at site 1, and will likely further stabilize the folded form of the PK helix, so locking the riboswitch in the translationally OFF state.

Finally it is instructive to compare the binding of the nicotinamide moiety of NAD^+^ and NMN by the NAD^+^-II riboswitch to that observed in proteins. In the riboswitch both ligands are extensively hydrogen bonded, and stacked with nucleobases on one (site 2) or both (site 1) sides. Some examples of enzymes that bind NAD^+^ or NMN are shown in Supplementary Figure S7. Some are bound via a number of hydrogen bonds to the protein. For example liver alcohol dehydrogenase (Supplementary Figure S7A) makes three hydrogen bonds from the amide and one from each of the ribose hydroxyl groups of the nicotinamide moiety of NAD^+^ (25) (PDB ID 2JHF). This is comparable to NMN bound to site 1 of the NAD^+^-II riboswitch. However, the pyridyl ring of the NAD^+^ is not stacked with aromatic rings of the protein on either side. By contrast in structures of NMN bound to *H. influenzae* acid phosphatase (26) (PDB ID 3OCU) (Supplementary Figure S7B) and human nicotinamide phosphoribosyltransferase (Supplementary Figure S7C) (PDB ID 6TAC) the pyridyl ring is stacked between the phenylalanine and tyrosine rings in both cases. Yet the nicotinamide moiety makes only two hydrogen bonds in the first structure (ribose OH and phosphate) and just a single hydrogen bond in the second (amide N). By contrast, NMN bound to site 1 of the NAD^+^-II riboswitch makes a total of five hydrogen bonds to the RNA, and the pyridyl ring is stacked with nucleobases on both faces. Thus the RNA maximises its interaction with the ligand, underlining that RNA can be an excellent receptor for small molecules.

## DATA AVAILABILITY

The coordinates and structure factors of all the reported crystal structures have been deposited in the PDB under accession numbers 8HB1 and 8I3Z (two strands with NMN), 8HB3 (two strands with NR), 8HB8 (single strand with NMN), 8HBA (single strand with NAD^+^).

## Supplementary Material

gkad102_Supplemental_Files
