# Supplementary material for: Crystal structures of the NAD+-II riboswitch reveal two distinct ligand-binding pockets
Source: Nucleic Acids Res. 2023 Feb 25;51(6):2904–14. doi: 10.1093/nar/gkad102 (PMC10085692; doi:10.1093/nar/gkad102)
Supplement: gkad102_Supplemental_Files [file gkad102_supplemental_files.zip › 8I3Z_val-report-full_P1.pdf]

Full wwPDB X-ray Structure Validation Report (i)

Jan 27, 2023 – 02:16 PM JST

PDB ID	:	8I3Z
Title	:	Crystal structure of NAD-II riboswitch (two strands) with NMN at 1.67 angstrom
Deposited on	:	2023-01-18
Resolution	:	1.67 Å(reported)

This wwPDB validation report is for manuscript review

For Manuscript Review

This is a Full wwPDB X-ray Structure Validation Report.

This report is produced by the wwPDB biocuration pipeline after annotation of the structure.

We welcome your comments at *validation@mail.wwpdb.org*
A user guide is available at
https://www.wwpdb.org/validation/2017/XrayValidationReportHelp
with specific help available everywhere you see the (i) symbol.

The types of validation reports are described at
http://www.wwpdb.org/validation/2017/FAQs#types.

The following versions of software and data (see references (i)) were used in the production of this report:

MolProbity	:	4.02b-467
Mogul	:	1.8.5 (274361), CSD as541be (2020)
Xtriage (Phenix)	:	1.13
EDS	:	2.31.3
buster-report	:	1.1.7 (2018)
Percentile statistics	:	20191225.v01 (using entries in the PDB archive December 25th 2019)
Refmac	:	5.8.0158
CCP4	:	7.0.044 (Gargrove)
Ideal geometry (proteins)	:	Engh & Huber (2001)

1 Overall quality at a glance (i)

The following experimental techniques were used to determine the structure:
X-RAY DIFFRACTION

The reported resolution of this entry is 1.67 Å.

Percentile scores (ranging between 0-100) for global validation metrics of the entry are shown in the following graphic. The table shows the number of entries on which the scores are based.

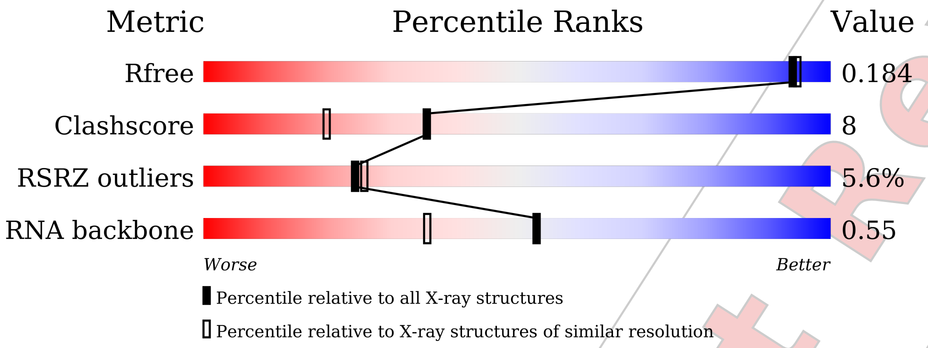

Metric	Whole archive (#Entries)	Similar resolution (#Entries, resolution range(Å))
R_{free}	130704	6780 (1.70-1.66)
Clashscore	141614	7310 (1.70-1.66)
RSRZ outliers	127900	6661 (1.70-1.66)
RNA backbone	3102	1005 (2.36-1.00)

The table below summarises the geometric issues observed across the polymeric chains and their fit to the electron density. The red, orange, yellow and green segments of the lower bar indicate the fraction of residues that contain outliers for >=3, 2, 1 and 0 types of geometric quality criteria respectively. A grey segment represents the fraction of residues that are not modelled. The numeric value for each fraction is indicated below the corresponding segment, with a dot representing fractions <=5% The upper red bar (where present) indicates the fraction of residues that have poor fit to the electron density. The numeric value is given above the bar.

Mol	Chain	Length	Quality of chain
1	A	24	58% 33% • •
2	B	31	10% / 39% 48% 13%

Ideal geometry (DNA, RNA) : Parkinson et al. (1996)
Validation Pipeline (wwPDB-VP) : 2.31.3

For Manuscript Review

2 Entry composition (i)

There are 5 unique types of molecules in this entry. The entry contains 1975 atoms, of which 603 are hydrogens and 0 are deuteriums.

In the tables below, the ZeroOcc column contains the number of atoms modelled with zero occupancy, the AltConf column contains the number of residues with at least one atom in alternate conformation and the Trace column contains the number of residues modelled with at most 2 atoms.

- Molecule 1 is a RNA chain called RNA (5'-R(*AP*GP*AP*GP*CP*GP*UP*UP*GP*CP*GP*UP*CP*CP*GP*AP*AP*AP*GP*UP*(CBV)P*GP*CP*C)-3').

Mol	Chain	Residues	Atoms							ZeroOcc	AltConf	Trace
			Total	Br	C	H	N	O	P			
1	A	24	772	1	229	260	94	165	23	0	0	0

- Molecule 2 is a RNA chain called RNA (31-MER).

Mol	Chain	Residues	Atoms						ZeroOcc	AltConf	Trace
			Total	C	H	N	O	P			
2	B	31	981	300	315	131	205	30	0	0	0

- Molecule 3 is SODIUM ION (three-letter code: NA) (formula: Na).

Mol	Chain	Residues	Atoms		ZeroOcc	AltConf
			Total	Na		
3	A	1	1	1	0	0
3	B	3	3	3	0	0

- Molecule 4 is BETA-NICOTINAMIDE RIBOSE MONOPHOSPHATE (three-letter code: NMN) (formula: $C_{11}H_{16}N_2O_8P$) (labeled as "Ligand of Interest" by depositor).

For Manuscript Review

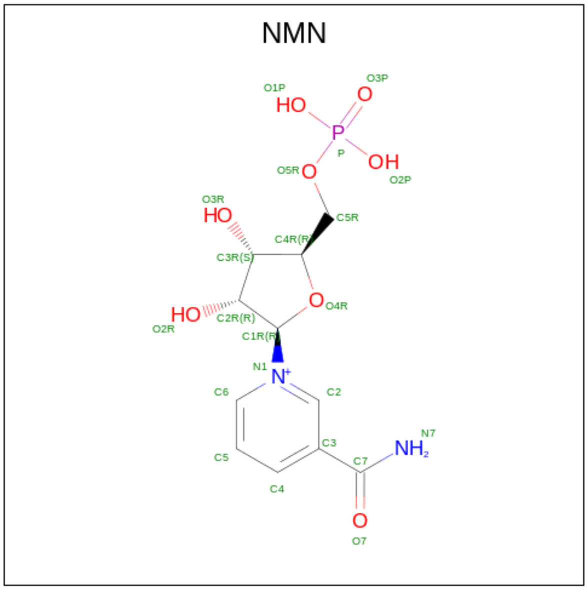

Mol	Chain	Residues	Atoms	ZeroOcc	AltConf
4	B	1	Total C H N O P<br>36 11 14 2 8 1	0	0
4	B	1	Total C H N O P<br>36 11 14 2 8 1	0	0

- Molecule 5 is water.

Mol	Chain	Residues	Atoms	ZeroOcc	AltConf
5	A	60	Total O<br>60 60	0	0
5	B	86	Total O<br>86 86	0	0

For Manuscript Review

3 Residue-property plots (i)

These plots are drawn for all protein, RNA, DNA and oligosaccharide chains in the entry. The first graphic for a chain summarises the proportions of the various outlier classes displayed in the second graphic. The second graphic shows the sequence view annotated by issues in geometry and electron density. Residues are color-coded according to the number of geometric quality criteria for which they contain at least one outlier: green = 0, yellow = 1, orange = 2 and red = 3 or more. A red dot above a residue indicates a poor fit to the electron density (RSRZ > 2). Stretches of 2 or more consecutive residues without any outlier are shown as a green connector. Residues present in the sample, but not in the model, are shown in grey.

• Molecule 1: RNA (5'-R(*AP*GP*AP*GP*CP*GP*UP*UP*GP*CP*GP*UP*CP*CP*GP*AP *AP*AP*GP*UP*(CBV)P*GP*CP*C)-3')

Chain A:

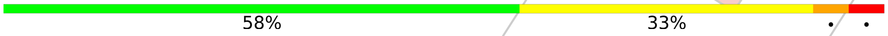

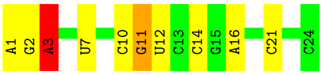

• Molecule 2: RNA (31-MER)

Chain B:

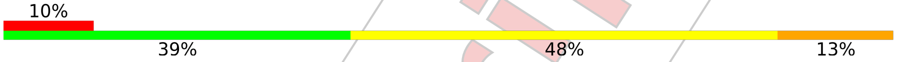

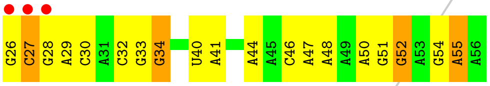

For Manuscript Review

4 Data and refinement statistics (i)

Property	Value	Source
Space group	P 32 2 1	Depositor
Cell constants<br>a, b, c, α, β, γ	81.27Å 81.27Å 61.85Å<br>90.00° 90.00° 120.00°	Depositor
Resolution (Å)	30.59 – 1.67<br>46.46 – 1.67	Depositor<br>EDS
% Data completeness<br>(in resolution range)	99.0 (30.59-1.67)<br>99.1 (46.46-1.67)	Depositor<br>EDS
R_{merge}	0.06	Depositor
R_{sym}	(Not available)	Depositor
$< I/\sigma(I) >$ [1]	1.85 (at 1.67Å)	Xtriage
Refinement program	PHENIX dev_4788, PHENIX dev_4788	Depositor
R, R_{free}	0.168 , 0.186<br>0.167 , 0.184	Depositor<br>DCC
R_{free} test set	1370 reflections (4.98%)	wwPDB-VP
Wilson B-factor (Å^2)	24.8	Xtriage
Anisotropy	0.497	Xtriage
Bulk solvent k_{sol}(e/Å^3), B_{sol}(Å^2)	0.36 , 50.1	EDS
L-test for twinning[2]	$< \vert L \vert > = 0.51$, $< L^2 > = 0.34$	Xtriage
Estimated twinning fraction	0.037 for -h,-k,l	Xtriage
F_o,F_c correlation	0.96	EDS
Total number of atoms	1975	wwPDB-VP
Average B, all atoms (Å^2)	46.0	wwPDB-VP

Xtriage's analysis on translational NCS is as follows: *The largest off-origin peak in the Patterson function is 4.95% of the height of the origin peak. No significant pseudotranslation is detected.*

[1] Intensities estimated from amplitudes.
[2] Theoretical values of $< |L| >$, $< L^2 >$ for acentric reflections are 0.5, 0.333 respectively for untwinned datasets, and 0.375, 0.2 for perfectly twinned datasets.

For Manuscript Review

5 Model quality (i)

5.1 Standard geometry (i)

Bond lengths and bond angles in the following residue types are not validated in this section: NA, CBV, NMN

The Z score for a bond length (or angle) is the number of standard deviations the observed value is removed from the expected value. A bond length (or angle) with $|Z| > 5$ is considered an outlier worth inspection. RMSZ is the root-mean-square of all Z scores of the bond lengths (or angles).

Mol	Chain	Bond lengths		Bond angles	
		RMSZ	$\#\|Z\|>5$	RMSZ	$\#\|Z\|>5$
1	A	1.34	6/548 (1.1%)	1.85	18/851 (2.1%)
2	B	1.17	3/748 (0.4%)	1.69	21/1165 (1.8%)
All	All	1.25	9/1296 (0.7%)	1.76	39/2016 (1.9%)

All (9) bond length outliers are listed below:

Mol	Chain	Res	Type	Atoms	Z	Observed(Å)	Ideal(Å)
1	A	3	A	P-O5'	-7.16	1.52	1.59
1	A	10	C	N1-C6	-6.29	1.33	1.37
2	B	48	A	N3-C4	5.44	1.38	1.34
1	A	2	G	C6-N1	-5.43	1.35	1.39
2	B	52	G	C6-N1	-5.33	1.35	1.39
2	B	52	G	N3-C4	-5.30	1.31	1.35
1	A	3	A	O5'-C5'	-5.29	1.34	1.42
1	A	1	A	N3-C4	5.29	1.38	1.34
1	A	11	G	C8-N7	-5.02	1.27	1.30

All (39) bond angle outliers are listed below:

Mol	Chain	Res	Type	Atoms	Z	Observed(°)	Ideal(°)
1	A	3	A	O5'-P-OP1	-14.39	92.75	105.70
1	A	11	G	O5'-P-OP2	-10.78	96.00	105.70
1	A	1	A	C2-N3-C4	-10.69	105.25	110.60
1	A	11	G	C5-C6-O6	9.54	134.33	128.60
2	B	50	A	N1-C2-N3	-7.77	125.42	129.30
1	A	11	G	C2-N3-C4	-7.53	108.14	111.90
1	A	3	A	C5'-C4'-C3'	-7.26	104.38	116.00
2	B	47	A	N1-C6-N6	7.11	122.87	118.60
1	A	3	A	C5-N7-C8	-7.07	100.37	103.90
2	B	52	G	C2-N3-C4	-7.01	108.39	111.90

Continued on next page...

For Manuscript Review

Continued from previous page...

Mol	Chain	Res	Type	Atoms	Z	Observed(°)	Ideal(°)
1	A	1	A	C5-C6-N1	-6.95	114.22	117.70
1	A	3	A	O5'-P-OP2	6.88	118.96	110.70
1	A	3	A	C2-N3-C4	-6.78	107.21	110.60
2	B	55	A	O5'-P-OP2	-6.29	100.03	105.70
1	A	7	U	C5-C6-N1	-6.28	119.56	122.70
1	A	1	A	N1-C2-N3	6.21	132.41	129.30
1	A	11	G	N1-C6-O6	-6.21	116.17	119.90
2	B	52	G	N3-C4-C5	6.18	131.69	128.60
2	B	46	C	N3-C2-O2	6.12	126.18	121.90
2	B	47	A	C5-N7-C8	-5.80	101.00	103.90
2	B	51	G	C8-N9-C4	5.77	108.71	106.40
2	B	51	G	N3-C4-C5	5.77	131.49	128.60
1	A	7	U	C6-N1-C2	5.75	124.45	121.00
2	B	54	G	C2-N3-C4	-5.73	109.04	111.90
1	A	3	A	N7-C8-N9	5.67	116.64	113.80
2	B	34	G	C5-C6-O6	-5.60	125.24	128.60
1	A	14	C	C6-N1-C2	5.54	122.52	120.30
2	B	34	G	C4-C5-N7	5.48	112.99	110.80
2	B	32	C	O5'-P-OP2	-5.40	100.84	105.70
1	A	14	C	C5-C6-N1	-5.37	118.32	121.00
2	B	50	A	C2-N3-C4	5.33	113.27	110.60
2	B	44	A	O4'-C1'-N9	5.31	112.44	108.20
1	A	11	G	OP1-P-OP2	5.30	127.56	119.60
2	B	27	C	C6-N1-C2	-5.26	118.20	120.30
2	B	52	G	N3-C4-N9	-5.20	122.88	126.00
2	B	46	C	N1-C2-O2	-5.19	115.79	118.90
2	B	32	C	N3-C4-C5	5.17	123.97	121.90
2	B	40	U	O5'-P-OP1	5.17	116.90	110.70
2	B	41	A	O4'-C1'-N9	5.01	112.21	108.20

There are no chirality outliers.

There are no planarity outliers.

5.2 Too-close contacts ⓘ

In the following table, the Non-H and H(model) columns list the number of non-hydrogen atoms and hydrogen atoms in the chain respectively. The H(added) column lists the number of hydrogen atoms added and optimized by MolProbity. The Clashes column lists the number of clashes within the asymmetric unit, whereas Symm-Clashes lists symmetry-related clashes.

For Manuscript Review

Mol	Chain	Non-H	H(model)	H(added)	Clashes	Symm-Clashes
1	A	512	260	261	4	0
2	B	666	315	339	11	0
3	A	1	0	0	0	0
3	B	3	0	0	0	0
4	B	44	28	27	2	0
5	A	60	0	0	0	0
5	B	86	0	0	0	0
All	All	1372	603	627	15	0

The all-atom clashscore is defined as the number of clashes found per 1000 atoms (including hydrogen atoms). The all-atom clashscore for this structure is 8.

All (15) close contacts within the same asymmetric unit are listed below, sorted by their clash magnitude.

Atom-1	Atom-2	Interatomic distance (Å)	Clash overlap (Å)
2:B:26:G:H2'	2:B:27:C:H5'	1.56	0.88
1:A:3:A:H8	1:A:3:A:H5"	1.38	0.86
2:B:26:G:C2'	2:B:27:C:H5'	2.09	0.82
2:B:26:G:H2'	2:B:27:C:C5'	2.18	0.72
1:A:3:A:H5"	1:A:3:A:C8	2.24	0.71
2:B:26:G:O2'	2:B:27:C:H5'	1.97	0.65
2:B:26:G:C2'	2:B:27:C:C5'	2.82	0.57
1:A:3:A:C8	1:A:3:A:C5'	2.91	0.53
2:B:29:A:O2'	2:B:30:C:H5'	2.10	0.52
2:B:29:A:C2'	2:B:30:C:H5'	2.41	0.51
1:A:3:A:H8	1:A:3:A:C5'	2.18	0.47
2:B:33:G:H2'	2:B:34:G:O4'	2.15	0.46
2:B:52:G:O6	4:B:102:NMN:HC6	2.17	0.44
2:B:27:C:O2'	2:B:28:G:OP2	2.27	0.43
2:B:33:G:O6	4:B:101:NMN:HC2	2.21	0.40

There are no symmetry-related clashes.

5.3 Torsion angles (i)

5.3.1 Protein backbone (i)

There are no protein molecules in this entry.

For Manuscript Review

5.3.2 Protein sidechains (i)

There are no protein molecules in this entry.

5.3.3 RNA (i)

Mol	Chain	Analysed	Backbone Outliers	Pucker Outliers
1	A	22/24 (91%)	4 (18%)	1 (4%)
2	B	30/31 (96%)	1 (3%)	0
All	All	52/55 (94%)	5 (9%)	1 (1%)

All (5) RNA backbone outliers are listed below:

Mol	Chain	Res	Type
1	A	3	A
1	A	11	G
1	A	12	U
1	A	16	A
2	B	55	A

All (1) RNA pucker outliers are listed below:

Mol	Chain	Res	Type
1	A	11	G

5.4 Non-standard residues in protein, DNA, RNA chains (i)

1 non-standard protein/DNA/RNA residue is modelled in this entry.

In the following table, the Counts columns list the number of bonds (or angles) for which Mogul statistics could be retrieved, the number of bonds (or angles) that are observed in the model and the number of bonds (or angles) that are defined in the Chemical Component Dictionary. The Link column lists molecule types, if any, to which the group is linked. The Z score for a bond length (or angle) is the number of standard deviations the observed value is removed from the expected value. A bond length (or angle) with $|Z| > 2$ is considered an outlier worth inspection. RMSZ is the root-mean-square of all Z scores of the bond lengths (or angles).

Mol	Type	Chain	Res	Link	Bond lengths			Bond angles		
					Counts	RMSZ	$\#\|Z\| > 2$	Counts	RMSZ	$\#\|Z\| > 2$
1	CBV	A	21	2,1	19,22,23	3.27	8 (42%)	27,32,35	0.81	1 (3%)

In the following table, the Chirals column lists the number of chiral outliers, the number of chiral centers analysed, the number of these observed in the model and the number defined in the

For Manuscript Review

Chemical Component Dictionary. Similar counts are reported in the Torsion and Rings columns. '-' means no outliers of that kind were identified.

Mol	Type	Chain	Res	Link	Chirals	Torsions	Rings
1	CBV	A	21	2,1	-	0/7/25/26	0/2/2/2

All (8) bond length outliers are listed below:

Mol	Chain	Res	Type	Atoms	Z	Observed(Å)	Ideal(Å)
1	A	21	CBV	C6-C5	7.31	1.48	1.34
1	A	21	CBV	C4-N3	5.98	1.44	1.34
1	A	21	CBV	C2-N3	5.95	1.48	1.36
1	A	21	CBV	C4-N4	4.48	1.45	1.34
1	A	21	CBV	C6-N1	3.97	1.44	1.38
1	A	21	CBV	O2-C2	-3.80	1.16	1.23
1	A	21	CBV	C2-N1	3.80	1.48	1.40
1	A	21	CBV	C4-C5	2.99	1.49	1.42

All (1) bond angle outliers are listed below:

Mol	Chain	Res	Type	Atoms	Z	Observed(^{o})	Ideal(^{o})
1	A	21	CBV	C1'-N1-C6	-2.13	117.58	121.12

There are no chirality outliers.

There are no torsion outliers.

There are no ring outliers.

No monomer is involved in short contacts.

5.5 Carbohydrates (i)

There are no monosaccharides in this entry.

5.6 Ligand geometry (i)

Of 6 ligands modelled in this entry, 4 are monoatomic - leaving 2 for Mogul analysis.

In the following table, the Counts columns list the number of bonds (or angles) for which Mogul statistics could be retrieved, the number of bonds (or angles) that are observed in the model and the number of bonds (or angles) that are defined in the Chemical Component Dictionary. The Link column lists molecule types, if any, to which the group is linked. The Z score for a bond length (or angle) is the number of standard deviations the observed value is removed from the

For Manuscript Review

expected value. A bond length (or angle) with $|Z| > 2$ is considered an outlier worth inspection. RMSZ is the root-mean-square of all Z scores of the bond lengths (or angles).

Mol	Type	Chain	Res	Link	Bond lengths			Bond angles		
					Counts	RMSZ	#|Z| > 2	Counts	RMSZ	#|Z| > 2
4	NMN	B	101	-	22,23,23	3.44	8 (36%)	30,34,34	1.83	5 (16%)
4	NMN	B	102	-	22,23,23	3.24	10 (45%)	30,34,34	2.17	10 (33%)

In the following table, the Chirals column lists the number of chiral outliers, the number of chiral centers analysed, the number of these observed in the model and the number defined in the Chemical Component Dictionary. Similar counts are reported in the Torsion and Rings columns. '-' means no outliers of that kind were identified.

Mol	Type	Chain	Res	Link	Chirals	Torsions	Rings
4	NMN	B	101	-	-	1/14/30/30	0/2/2/2
4	NMN	B	102	-	-	0/14/30/30	0/2/2/2

All (18) bond length outliers are listed below:

Mol	Chain	Res	Type	Atoms	Z	Observed(Å)	Ideal(Å)
4	B	101	NMN	C3R-C4R	-8.93	1.30	1.53
4	B	102	NMN	C3R-C4R	-8.48	1.31	1.53
4	B	101	NMN	O4R-C4R	7.66	1.62	1.45
4	B	102	NMN	O4R-C4R	7.09	1.60	1.45
4	B	101	NMN	O4R-C1R	-5.86	1.32	1.41
4	B	101	NMN	C3-C7	5.24	1.58	1.50
4	B	102	NMN	O4R-C1R	-5.15	1.33	1.41
4	B	101	NMN	C7-N7	4.85	1.42	1.33
4	B	102	NMN	C7-N7	4.25	1.41	1.33
4	B	102	NMN	C2R-C1R	3.86	1.59	1.53
4	B	102	NMN	C2-N1	-3.53	1.30	1.35
4	B	102	NMN	C3-C7	3.41	1.55	1.50
4	B	101	NMN	C4-C3	-3.38	1.33	1.39
4	B	102	NMN	C4-C3	-2.35	1.35	1.39
4	B	102	NMN	C5-C4	-2.34	1.34	1.38
4	B	102	NMN	O3R-C3R	2.28	1.48	1.43
4	B	101	NMN	O3R-C3R	2.19	1.48	1.43
4	B	101	NMN	C2-C3	-2.17	1.35	1.39

All (15) bond angle outliers are listed below:

Mol	Chain	Res	Type	Atoms	Z	Observed(°)	Ideal(°)
4	B	101	NMN	C3-C7-N7	5.67	124.55	117.75
4	B	101	NMN	C2-C3-C4	4.62	123.49	118.26

Continued on next page...

For Manuscript Review

Continued from previous page...

Mol	Chain	Res	Type	Atoms	Z	Observed(°)	Ideal(°)
4	B	102	NMN	C5-C6-N1	-4.41	114.08	120.40
4	B	102	NMN	O4R-C4R-C5R	-4.37	94.99	109.37
4	B	102	NMN	O7-C7-N7	-4.36	116.38	122.58
4	B	102	NMN	C2R-C3R-C4R	4.26	110.91	102.64
4	B	102	NMN	C6-N1-C2	3.93	125.56	121.97
4	B	101	NMN	C3-C2-N1	-3.33	117.17	120.43
4	B	101	NMN	O7-C7-C3	-3.00	116.04	119.63
4	B	102	NMN	C3-C7-N7	2.85	121.17	117.75
4	B	102	NMN	C3R-C2R-C1R	-2.57	97.10	100.98
4	B	101	NMN	O7-C7-N7	-2.44	119.11	122.58
4	B	102	NMN	O3R-C3R-C2R	-2.23	104.61	111.82
4	B	102	NMN	O7-C7-C3	2.19	122.25	119.63
4	B	102	NMN	O2P-P-O5R	2.15	112.45	106.73

There are no chirality outliers.

All (1) torsion outliers are listed below:

Mol	Chain	Res	Type	Atoms
4	B	101	NMN	C5R-O5R-P-O1P

There are no ring outliers.

2 monomers are involved in 2 short contacts:

Mol	Chain	Res	Type	Clashes	Symm-Clashes
4	B	101	NMN	1	0
4	B	102	NMN	1	0

The following is a two-dimensional graphical depiction of Mogul quality analysis of bond lengths, bond angles, torsion angles, and ring geometry for all instances of the Ligand of Interest. In addition, ligands with molecular weight > 250 and outliers as shown on the validation Tables will also be included. For torsion angles, if less then 5% of the Mogul distribution of torsion angles is within 10 degrees of the torsion angle in question, then that torsion angle is considered an outlier. Any bond that is central to one or more torsion angles identified as an outlier by Mogul will be highlighted in the graph. For rings, the root-mean-square deviation (RMSD) between the ring in question and similar rings identified by Mogul is calculated over all ring torsion angles. If the average RMSD is greater than 60 degrees and the minimal RMSD between the ring in question and any Mogul-identified rings is also greater than 60 degrees, then that ring is considered an outlier. The outliers are highlighted in purple. The color gray indicates Mogul did not find sufficient equivalents in the CSD to analyse the geometry.

For Manuscript Review

Ligand NMN B 101

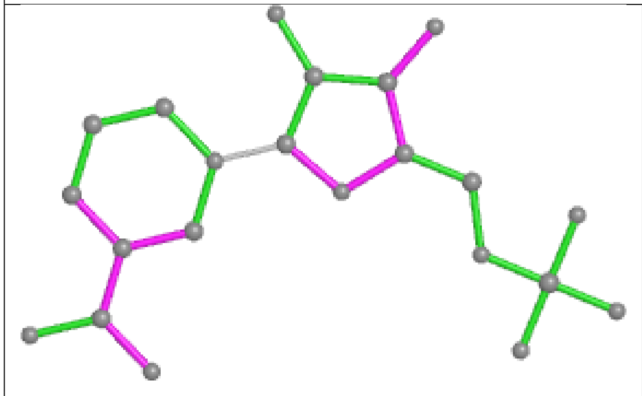
Bond lengths

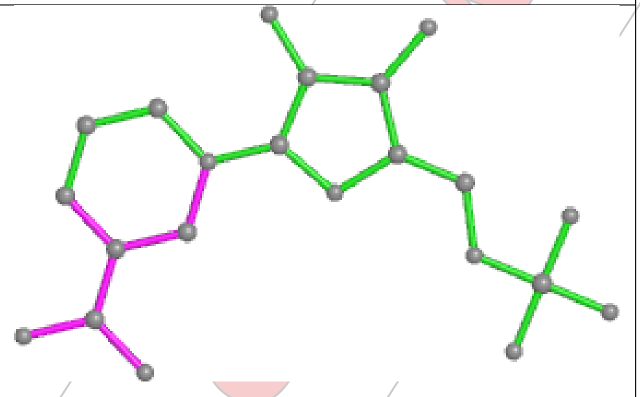
Bond angles

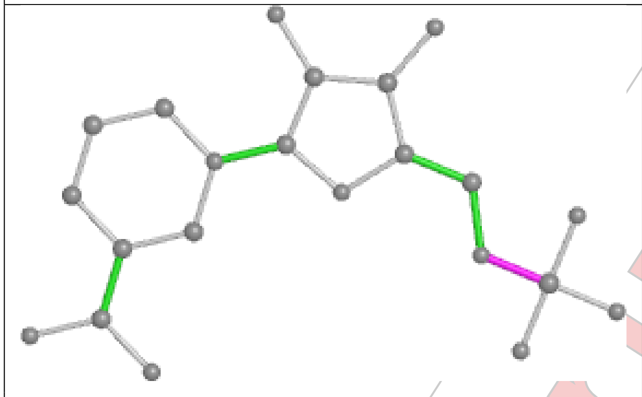
Torsions

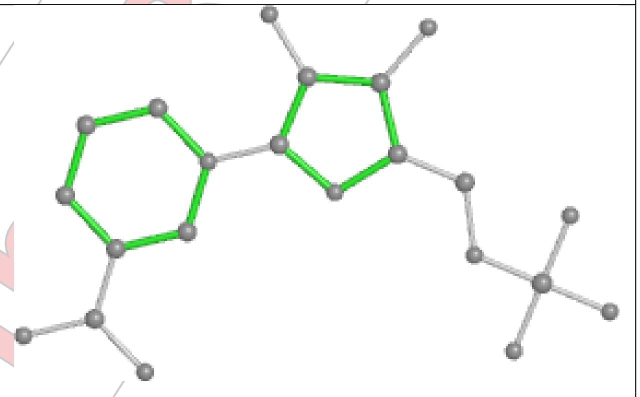
Rings

Ligand NMN B 102

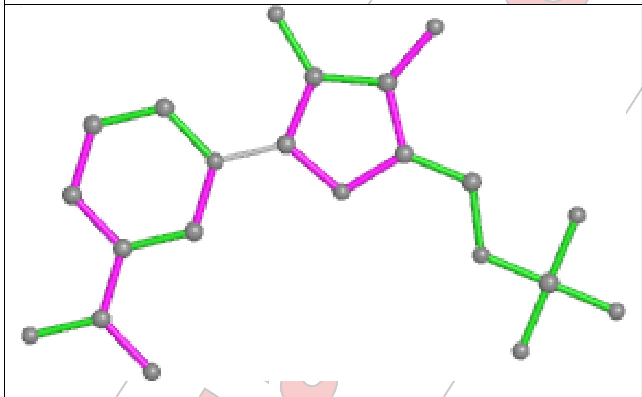
Bond lengths

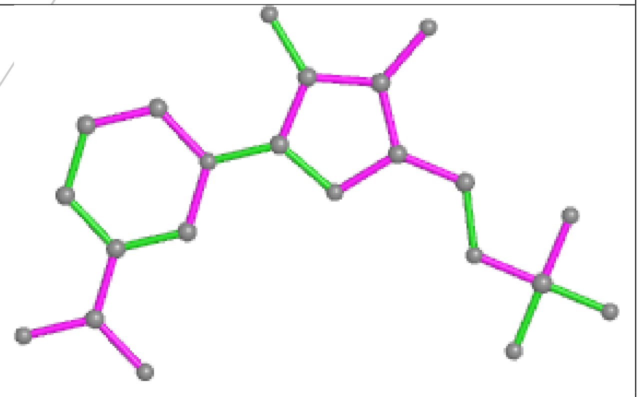
Bond angles

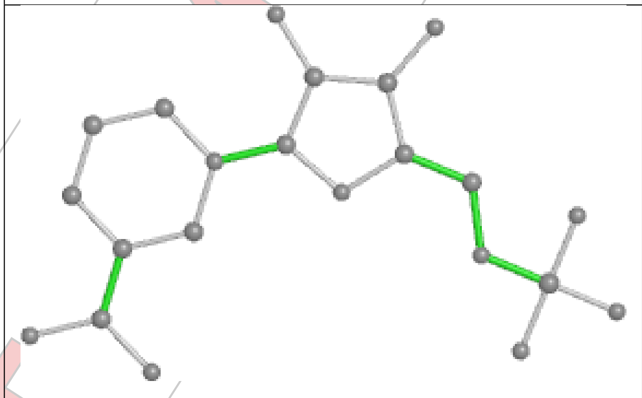
Torsions

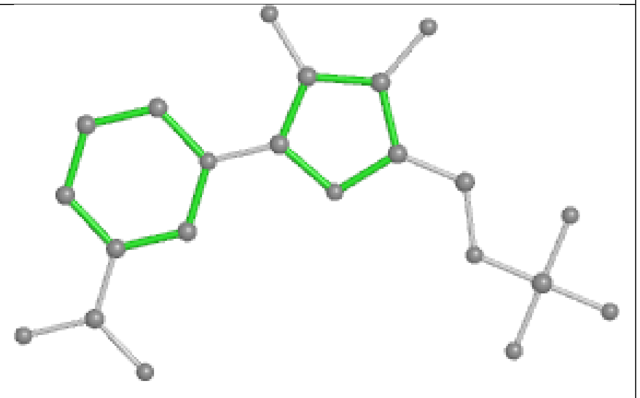
Rings

5.7 Other polymers (i)

There are no such residues in this entry.

5.8 Polymer linkage issues (i)

There are no chain breaks in this entry.

For Manuscript Review

6 Fit of model and data (i)

6.1 Protein, DNA and RNA chains (i)

In the following table, the column labelled '#RSRZ> 2' contains the number (and percentage) of RSRZ outliers, followed by percent RSRZ outliers for the chain as percentile scores relative to all X-ray entries and entries of similar resolution. The OWAB column contains the minimum, median, 95th percentile and maximum values of the occupancy-weighted average B-factor per residue. The column labelled 'Q< 0.9' lists the number of (and percentage) of residues with an average occupancy less than 0.9.

Mol	Chain	Analysed	<RSRZ>	#RSRZ>2			OWAB(Å^2)	Q<0.9
1	A	23/24 (95%)	-0.26	0	100	100	25, 31, 80, 112	0
2	B	31/31 (100%)	0.08	3 (9%)	7	8	26, 36, 103, 126	0
All	All	54/55 (98%)	-0.07	3 (5%)	24	25	25, 34, 103, 126	0

All (3) RSRZ outliers are listed below:

Mol	Chain	Res	Type	RSRZ
2	B	26	G	4.7
2	B	27	C	3.5
2	B	28	G	2.8

6.2 Non-standard residues in protein, DNA, RNA chains (i)

In the following table, the Atoms column lists the number of modelled atoms in the group and the number defined in the chemical component dictionary. The B-factors column lists the minimum, median, 95th percentile and maximum values of B factors of atoms in the group. The column labelled 'Q< 0.9' lists the number of atoms with occupancy less than 0.9.

Mol	Type	Chain	Res	Atoms	RSCC	RSR	B-factors(Å^2)	Q<0.9
1	CBV	A	21	21/22	0.98	0.07	55,66,77,84	0

6.3 Carbohydrates (i)

There are no monosaccharides in this entry.

6.4 Ligands (i)

In the following table, the Atoms column lists the number of modelled atoms in the group and the number defined in the chemical component dictionary. The B-factors column lists the minimum,

For Manuscript Review

median, 95th percentile and maximum values of B factors of atoms in the group. The column labelled 'Q< 0.9' lists the number of atoms with occupancy less than 0.9.

Mol	Type	Chain	Res	Atoms	RSCC	RSR	B-factors(Å^2)	Q<0.9
3	NA	B	104	1/1	0.62	0.14	63,63,63,63	0
3	NA	B	105	1/1	0.74	0.34	63,63,63,63	0
3	NA	B	103	1/1	0.94	0.08	47,47,47,47	0
3	NA	A	101	1/1	0.95	0.12	57,57,57,57	0
4	NMN	B	101	22/22	0.95	0.08	22,35,53,54	0
4	NMN	B	102	22/22	0.96	0.10	21,27,34,34	0

The following is a graphical depiction of the model fit to experimental electron density of all instances of the Ligand of Interest. In addition, ligands with molecular weight > 250 and outliers as shown on the geometry validation Tables will also be included. Each fit is shown from different orientation to approximate a three-dimensional view.

Electron density around NMN B 101:

2mF$_o$-DF$_c$ (at 0.7 rmsd) in gray
mF$_o$-DF$_c$ (at 3 rmsd) in purple (negative)
and green (positive)

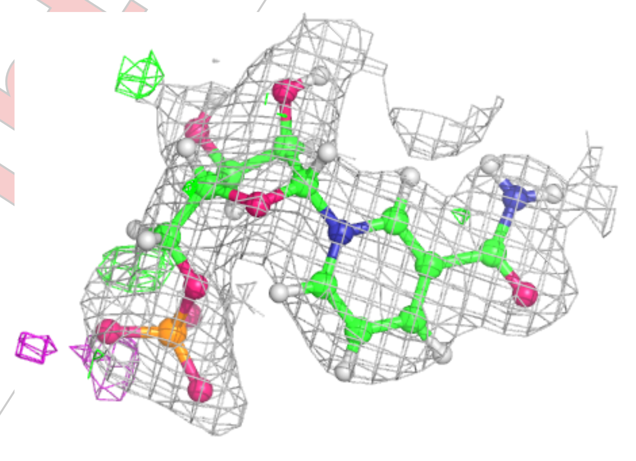

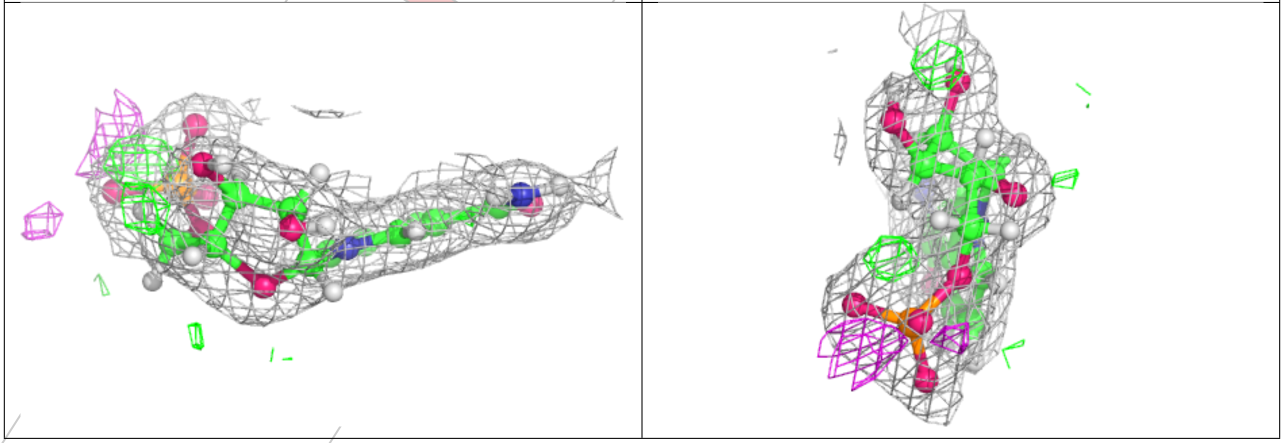

For Manuscript Review

Electron density around NMN B 102:

$2mF_o$-DF_c (at 0.7 rmsd) in gray
mF_o-DF_c (at 3 rmsd) in purple (negative)
and green (positive)

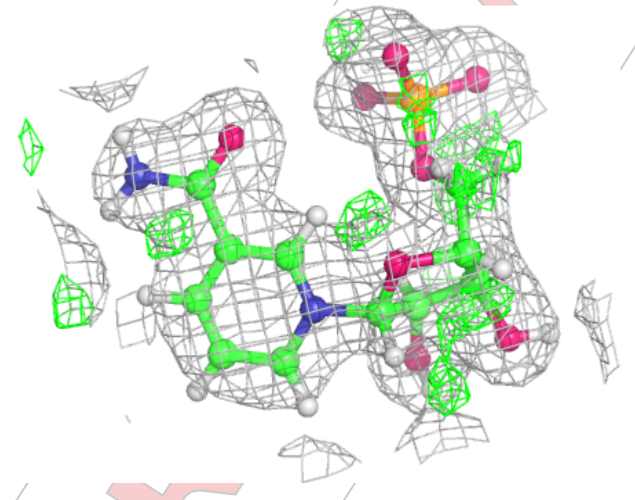

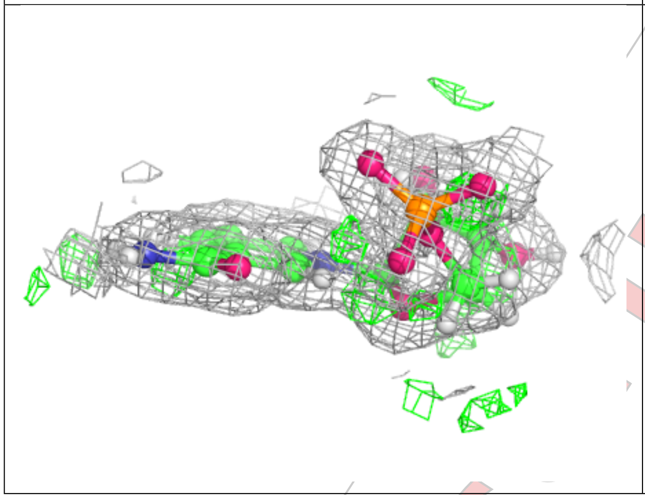

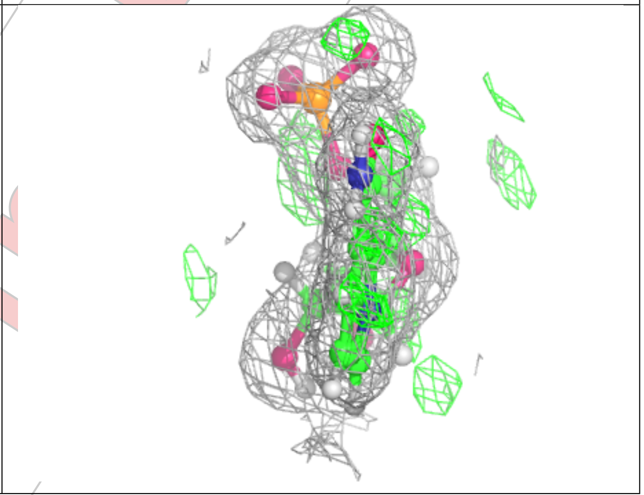

6.5 Other polymers (i)

There are no such residues in this entry.

For Manuscript Review
