# Supplementary material for: Crystal structures of the NAD+-II riboswitch reveal two distinct ligand-binding pockets
Source: Nucleic Acids Res. 2023 Feb 25;51(6):2904–14. doi: 10.1093/nar/gkad102 (PMC10085692; doi:10.1093/nar/gkad102)
Supplement: gkad102_Supplemental_Files [file gkad102_supplemental_files.zip › Peng_NAR3638_SI.pdf]

Crystal structures of the NAD$^+$-II riboswitch reveal two different ligand-binding pockets

Xuemei Peng, Wenjian Liao, Xiaowei Lin, David M. J. Lilley & Lin Huang

Supplementary Information

Supplementary Figures

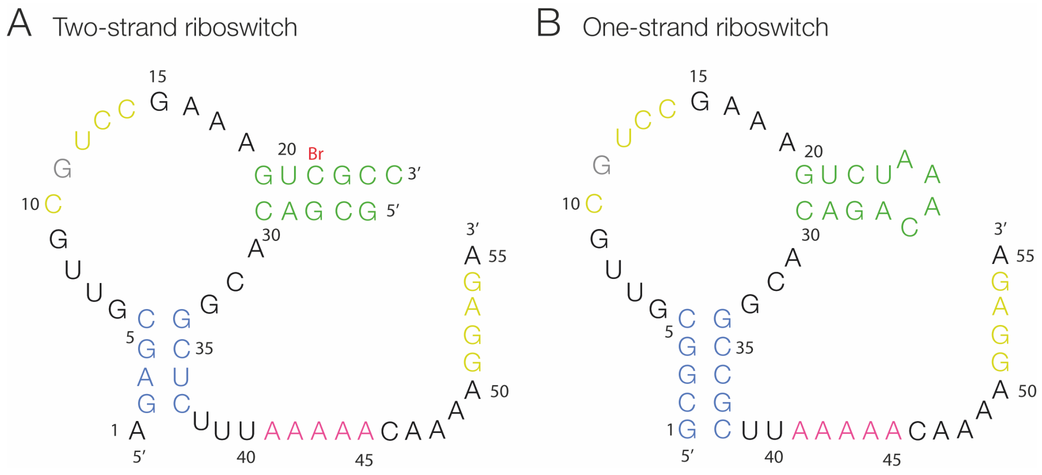

Supplementary Figure S1. Sequences of the two- and one-strand versions of the NAD$^+$-II riboswitch used for crystallization of complexes with bound ligands.

A. The two-strand riboswitch lacking the terminal loop in helix P1a. The strands were chemically synthesized, and the 5’ strand contains 5-bromocytidine at position 21, used to obtain crystallographic phase information.

B. The one-strand riboswitch with a terminal loop in helix P1a. This was generated by transcription using T7 RNA polymerase.

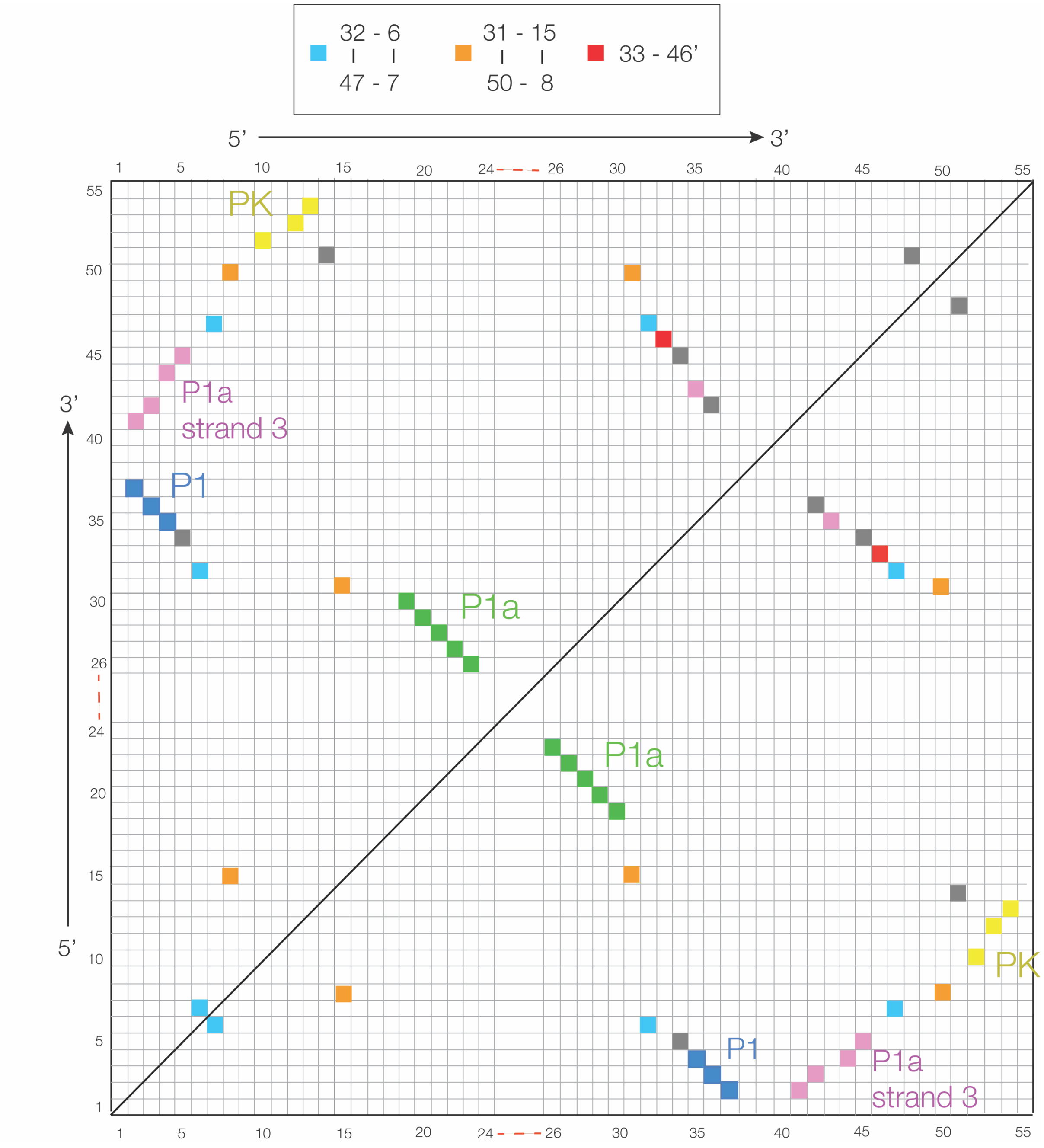

Supplementary Figure S2. Graph of nucleotide-nucleotide interactions. These are colored according to that used for the secondary structure throughout this work. The critical G33:C46 base pair in binding site 1 is colored red.

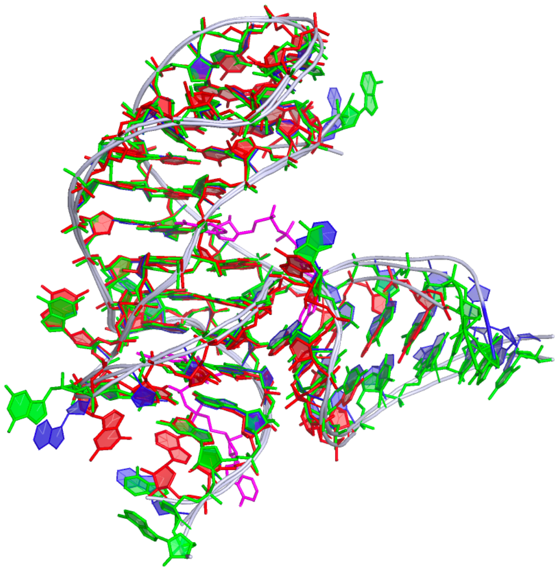

Supplementary Figure S3. Superposition of NAD⁺-II structures with different ligands bound as a parallel-eye stereoscopic image. The structures are : two-strand riboswitch with NMN bound (PDB ID 8HB1) colored blue ; two-strand riboswitch with NR bound (PDB ID 8HB3) colored green ; one-strand riboswitch with NAD⁺ bound (PDB ID 8HBA) colored red. The NAD⁺ ligand only is shown.

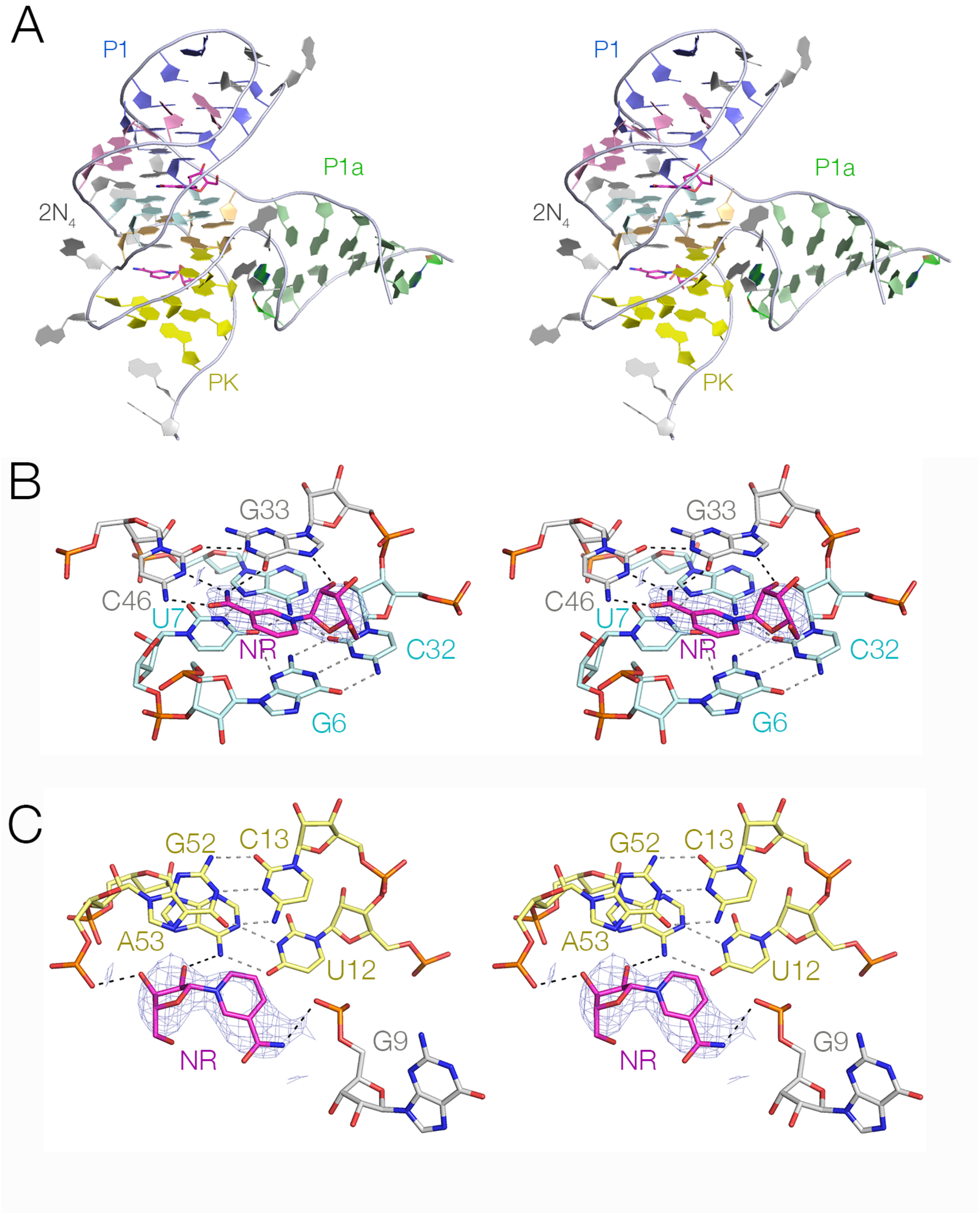

Supplementary Figure S4. The structure and ligand binding of the NAD⁺-II riboswitch with nicotinamide riboside. Parallel-eye stereoscopic views are shown. This follows the color scheme used throughout the work.

A. The overall structure of the riboswitch. The secondary structure elements are marked. The two NR ligands are shown in stick form, colored magenta.

B. NR bound at site 1 in the $2N_4$ core. An simulated annealing omit map contoured at 1.5 σ is shown on the NR ligand.

C. NR bound at site 2 in the major groove of the PK helix. A simulated annealing omit map contoured at 1.5 σ is shown on the NR ligand.

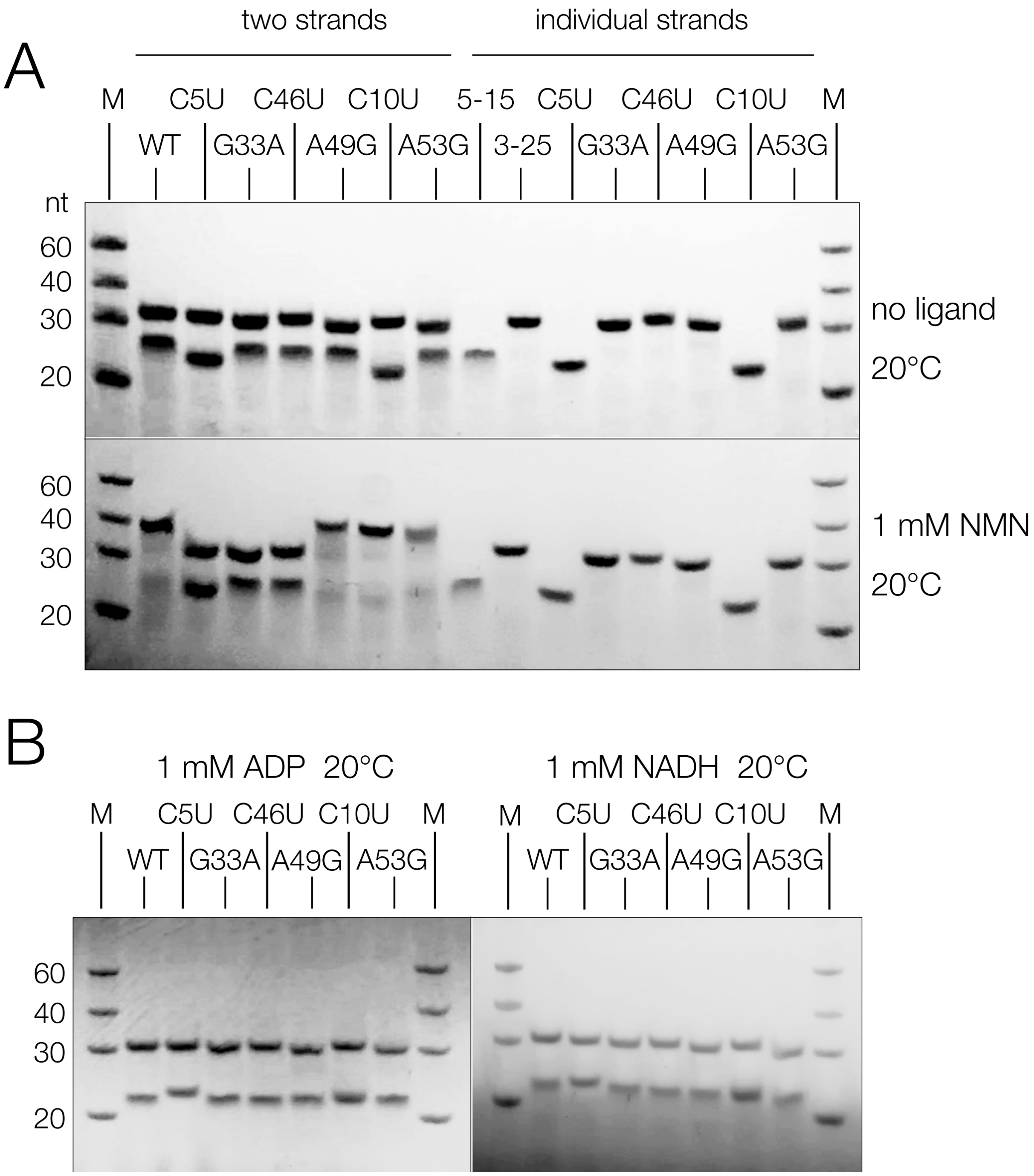

Supplementary Figure S5. Ligand binding by the NAD+-II riboswitch analysed by polyacrylamide gel electrophoresis at room temperature.

A. Polyacrylamide gel electrophoresis of wild-type and mutant NAD+-II riboswitches and their component individual strands in the absence of ligand (upper) or the presence of 1 mM NMN (lower).

B. Polyacrylamide gel electrophoresis of wild-type and mutant NAD+-II riboswitches in the presence of 1 mM ADP (left) or NADH (right). Note that neither compound leads to formation of a retarded single species, i.e. achieves formation of the folded riboswitch.

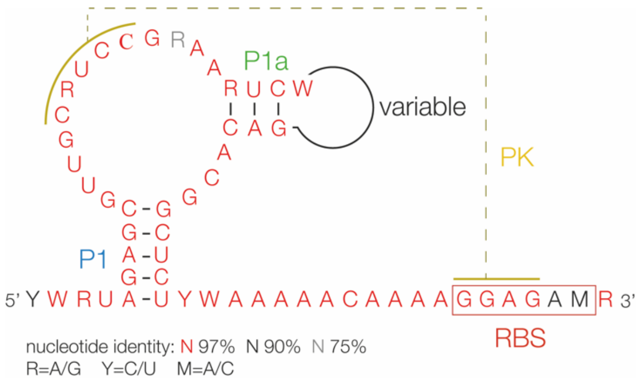

Supplementary Figure S6. Nucleotide conservation in the NAD^{+}-II riboswitch. These data are taken from Panchapakesan et al, *RNA* **27**, 99-105 (2021).

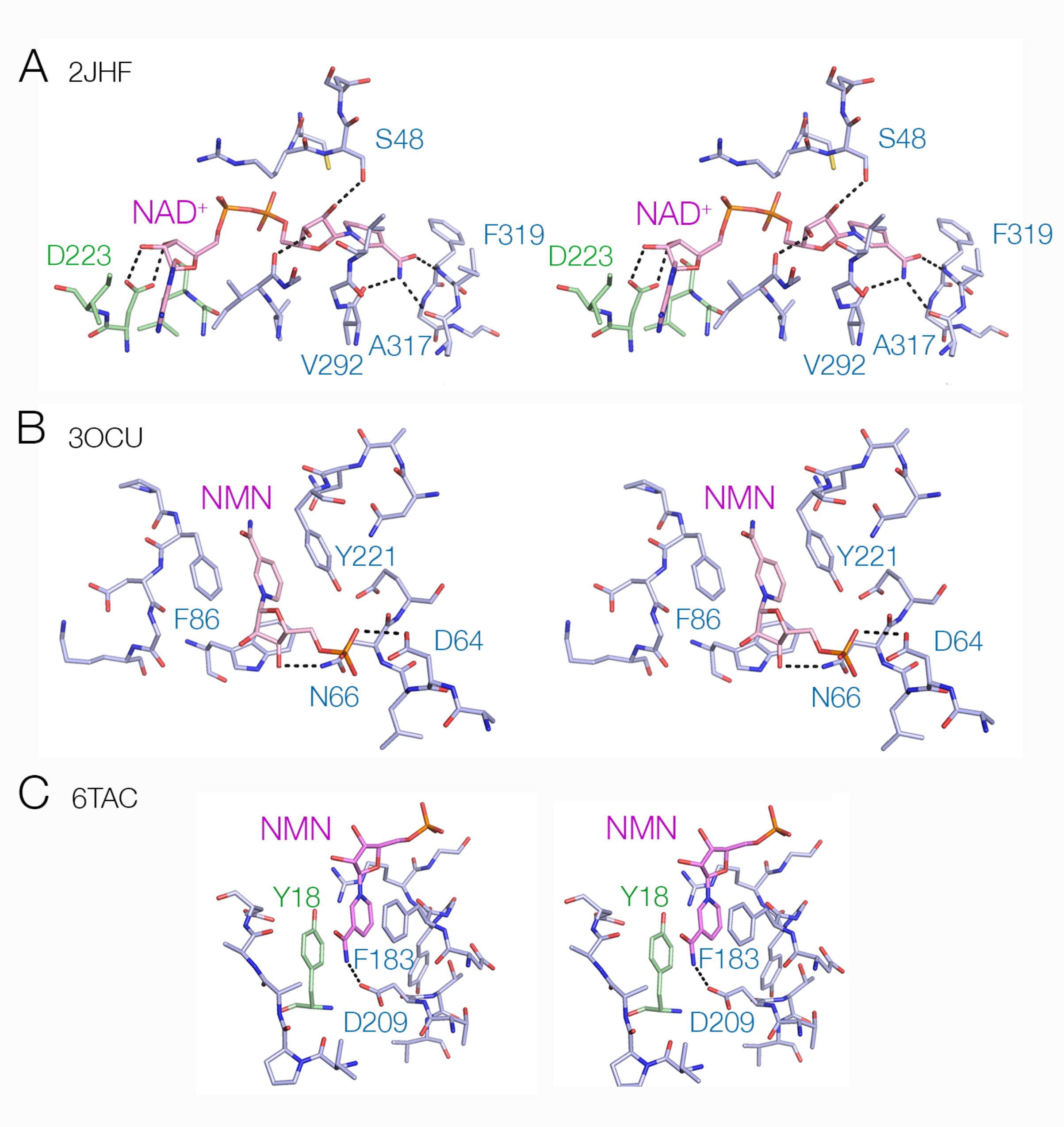

Supplementary Figure S7. Examples of nicotinamide binding to some proteins. Three examples of structures of nicotinamide-containing ligands bound to enzymes taken from the Protein Data Base.

A. NAD^+ bound to horse liver alcohol dehydrogenase. The nicotinamide moiety is extensively hydrogen bonded to the protein, but the pyridyl ring is not stacked with aromatic side chains. Amino acids colored green interact with the adenine moiety of NAD^+. Taken from PDB ID 2JHF.

B. NMN bound to *H. influenzae* acid phosphatase. The nicotinamide moiety is stacked between phenylalanine and tyrosine side chains, but the amide is not hydrogen bonded to the protein. Taken from PDB ID 3OCU.

C. NMN bound to human nicotinamide phosphoribosyltransferase. The nicotinamide moiety is stacked between phenylalanine and tyrosine side chains, but the amide only donates a single hydrogen bond. The tyrosine (Y18) colored green is part of a different polypeptide. Taken from PDB ID 6TAC.

Supplementary Tables

	crystallized with	mother liquor	cryo	resol^n	spacegroup	phasing
8HB1	two strands NMN	2.0 M ammonium sulfate	30% glycerol	2.23	P 3_2 2 1	SAD-Br FOM=0.192
	5-24	AGAGCGUUGCGUCCGAAAGU(BrC)GCC				
	3-30	GCGACACGGCUCUUUAAAAACAAAAGGAGA				
	S.par-55	AGAGCGUUGCGUCCGAAAGUCUAAACAGACACGGCUCUUUAAAAACAAAAGGAGA				
8I3Z	two strands NMN	2.0 M ammonium sulfate	30% glycerol	1.67	P 3_2 2 1	MR Model: 8HB1 TFZ=24.1 LLG=811
	5-24	AGAGCGUUGCGUCCGAAAGU(BrC)GCC				
	3-31	GCGACACGGCUCUUUAAAAACAAAAGGAGAA				
8HB3	two strands NR	2.0 M ammonium sulfate, soaking with 10 mM strontium chloride	30% glycerol	2.87	P 3_2 2 1	MR Model: 8HB1 TFZ=24.1 LLG=811
	5-24	AGAGCGUUGCGUCCGAAAGU(BrC)GCC				
	3-31	GCGACACGGCUCUUUAAAAACAAAAGGAGAA				
8HB8	single strand NMN	0.012 M sodium chloride,<br>0.08 M potassium chloride<br>0.04 M sodium cacodylate trihydrate pH 5.5<br>45% v/v (+/-)-2-methyl-2,4-pentanediol<br>0.02 M hexammine cobalt (III) chloride<br>soaking with barium chloride	direct	2.30	I 4_1 2 2	MR Model: 8HBA TFZ=20.5 LLG=358
	Template for transcription	TAATACGACTCACTATAGCGGCGTTGCGTCCGAAAGTCTAAACAGACACGGCCGCTTAAAAACAAAAGGAGAGGCCGGCATGCTCCCAGCCTCCTCGCGGCGCCGGCTGGGCAACTACTTCGGTAGGCGAATGGGATC				
	55	GCGGCGUUGCGUCCGAAAGUCUAAACAGACACGGCCGCUUAAAAACAAAAGGAGA				
8HBA	single strand NAD^+	0.012 M sodium chloride,<br>0.08 M potassium chloride<br>0.04 M sodium cacodylate trihydrate pH 5.5<br>45% v/v (+/-)-2-methyl-2,4-pentanediol<br>0.02 M hexammine cobalt (III) chloride	direct	2.64	P 2_1 2_1 2_1	MR Model: 8HB1 TFZ=26.7 LLG=829
	Template for transcription	TAATACGACTCACTATAGCGGCGTTGCGTCCGAAAGTCTAAACAGACACGGCCGCTTAAAAACAAAAGGAGAGGCCGGCATGCTCCCAGCCTCCTCGCGGCGCCGGCTGGGCAACTACTTCGGTAGGCGAATGGGATC				
	55	GCGGCGUUGCGUCCGAAAGUCUAAACAGACACGGCCGCUUAAAAACAAAAGGAGA				
	S.par-55	AGAGCGUUGCGUCCGAAAGUCUAAACAGACACGGCUCUUUAAAAACAAAAGGAGA				

Supplementary Table S1 Sequences and conditions employed in crystallization trials. *S.par*-55: *Streptococcus parasanguini pnu*C or 65 *pnu*C (Panchapakesan et al., *RNA* **27**, 99-105 (2021)). SAD-Br : single wavelength anomalous diffraction using bromocytosine. MR : molecular replacement. FOM : figure of merit. TFZ : translation function Z score. LLG : log-likelihood gain.

Name	Two strands NMN	Two strands NMN	Two strands NR	Single strand NMN	Single strand NAD^+
PDB	8HB1	8I3Z	8HB3	8HB8	8HBA
**Data collection**					
Space group	P 3_2 2 1	P 3_2 2 1	P 3_2 2 1	I 4_1 2 2	P 2_1 2_1 2_1
Cell dimensions					
a, *b*, *c* (Å)	82.3, 82.3, 63.2	81.3, 81.3, 61.9	83.0, 83.0, 65.6	121.4, 121.4, 109.1	37.8, 68.6, 114.0
α, β, γ (°)	90 90 120	90 90 120	90 90 120	90 90 90	90 90 90
	SAD-Br	MR	MR	MR	MR
Wavelength	0.9191	0.9791	0.9791	0.9785	0.9785
Resolution (Å)	47.31 – 2.23 (2.29 – 2.23)	46.46 – 1.67 (1.71 – 1.67)	65.63 – 2.87 (2.95 – 2.87)	50.00 – 2.30 (2.42 – 2.30)	19.60 – 2.64 (2.74 – 2.64)
R_{merge}	0.073 (0.849)	0.055 (0.962)	0.062 (1.312)	0.082 (3.410)	0.052 (0.90)
R_{pim}	0.017 (0.224)	0.014 (0.387)	0.015 (0.301)	0.017 (0.753)	0.016 (0.292)
$I / \sigma I$	22.4 (2.9)	22.6 (1.8)	23.2 (2.7)	26.6 (1.0)	23.3 (2.0)
CC (1/2)	0.99 (0.97)	1.00 (0.89)	1.00 (0.85)	1.00 (0.64)	1.00 (0.92)
Completeness (%)	100.0 (100)	99.2 (93.4)	100.0 (100)	93.2 (100)	99.2 (94.3)
Redundancy	19.2 (15.8)	16.0 (7.0)	19.0 (19.9)	25.0 (21.0)	12.0 (9.5)
**Refinement**					
Resolution (Å)	34.50 – 2.23 (2.31 – 2.23)	30.59 – 1.67 (1.73 – 1.67)	29.84 – 2.87 (2.97 – 2.87)	27.04 – 2.30 (2.38 – 2.30)	19.60 – 2.64 (2.74 – 2.64)
No. reflections	12387 (1203)	27501 (2563)	7538 (739)	17150 (1796)	9024 (843)
R_{work} / R_{free}	0.226 / 0.249	0.168 / 0.186	0.198 / 0.215	0.224 / 0.251	0.229 / 0.275
No. atoms					
macromolecules	1157	1178	1179	1218	2111
ligands	76	76	66	79	154
solvent	1	146	0	0	0
B-factors					
macromolecules	77.24	44.31	110.45	93.23	70.61
ligands	56.11	33.13	82.50	89.14	107.37
solvent	78.43				
R.m.s. deviations					
Bond lengths (Å)	0.009	0.012	0.005	0.002	0.009
Bond angles (°)	1.44	1.79	1.17	0.62	1.80

*Values in parentheses are for highest-resolution shell.

Supplementary Table S2. Details of data collection and refinement statistics for the crystallographic data as deposited with the PDB.
